# Investigation and application of a classical piecewise hybrid with a fractional derivative for the epidemic model: Dynamical transmission and modeling

**DOI:** 10.1371/journal.pone.0307732

**Published:** 2024-08-29

**Authors:** Muhammad Umer Saleem, Muhammad Farman, Kottakkaran Sooppy Nisar, Aqeel Ahmad, Zainab Munir, Evren Hincal

**Affiliations:** 1 Department of Mathematics, University of Education, Lahore, Pakistan; 2 Mathematics Research Center, Near East University, Nicosia, North Cyprus, Turkey; 3 Department of Computer Science and Mathematics, Lebanese American University, Beirut, Lebanon; 4 Department of Mathematics, College of Science and Humanities in Alkharj, Prince Sattam Bin Abdulaziz University, Alkharj, Saudi Arabia; 5 Saveetha School of Engineering, SIMATS, Chennai, India; 6 Department of Mathematics, Ghazi University, D G Khan, Pakistan; 7 Institute of Mathematics, Khwaja Fareed University of Engineering and Information Technology, Rahim Yar Khan, Pakistan; Institute of Space Technology, PAKISTAN

## Abstract

In this research, we developed an epidemic model with a combination of Atangana-Baleanu Caputo derivative and classical operators for the hybrid operator’s memory effects, allowing us to observe the dynamics and treatment effects at different time phases of syphilis infection caused by sex. The developed model properties, which take into account linear growth and Lipschitz requirements relating the rate of effects within its many sub-compartments according to the equilibrium points, include positivity, unique solution, exitance, and boundedness in the feasible domain. After conducting sensitivity analysis with various parameters influencing the model for the piecewise fractional operator, the reproductive number *R*_0_ for the biological viability of the model is determined. Generalized Ulam-Hyers stability results are employed to preserve global stability. The investigated model thus has a unique solution in the specified subinterval in light of the Banach conclusion, and contraction as a consequence holds for the Atangana-Baleanu Caputo derivative with classical operators. The piecewise model that has been suggested has a maximum of one solution. For numerical solutions, piecewise fractional hybrid operators at various fractional order values are solved using the Newton polynomial interpolation method. A comparison is also made between Caputo operator and the piecewise derivative proposed operator. This work improves our knowledge of the dynamics of syphilis and offers a solid framework for assessing the effectiveness of interventions for planning and making decisions to manage the illness.

## 1 Introduction

Mathematical models have become essential tools for analyzing the spread and control of infectious diseases. The process of formulating these models involves specifying assumptions, variables, and parameters, yielding conceptual results such as thresholds, basic reproduction numbers, contact numbers, and replacement numbers. Models, alongside computer simulations, serve as valuable experimental tools for developing and testing theories, evaluating quantitative hypotheses, addressing specific inquiries, assessing sensitivity to parameter changes, and estimating critical parameters from data. By comprehending the transmission dynamics of infectious diseases across communities, regions, and countries, more effective strategies can be devised to mitigate their spread. Mathematical models play pivotal roles in comparing, planning, implementing, evaluating, and optimizing various detection, prevention, therapy, and control programs. They also aid in designing and analyzing epidemiological surveys, suggesting essential data collection efforts, identifying trends, making general forecasts, and quantifying forecast uncertainties [1].

Three of the most well-known species that cause treponemal illnesses in humans include Treponema pallidum, Treponema pertenue, and Treponema carateum. It is not possible to identify the four members of the bacterial family using autoimmune, chemical, or physical techniques. Syphilis is the most easily contracted disease through sexual activity, while the other treponemal diseases are spread by direct contact with an infected person [2]. Usually, it is found on the genitalia, lips, tongue, buccal mucosa, anus, and fingers. For a few weeks, enlarged lymph node regions are also noted. They are pliable about the substrate, rigid, painless, free of packaging and necrosis, and their skin remains unaltered above [3]. Though they may develop at any stage of the disease, neurosyphilis, ocular syphilis, and otosyphilis are more likely to emerge in the early stages (primary, secondary, and early latent). Regardless of immunological condition, any patient can be affected by these complex syphilis infections. Individuals living with HIV infection, specifically those with low CD4 levels, have an increased risk of developing neurosyphilis and ocular syphilis quickly [4]. A newborn or fetus infected with Treponema pallidum during pregnancy from a mother who has untreated or insufficiently treated syphilis is said to have congenital syphilis. Infants infected with congenital syphilis may have physical and neurological issues for the rest of their lives, as well as miscarriage, stillbirth, or early infant mortality [5]. An untreated syphilis infection in humans advances through several phases. The exposed (infected but not yet infectious) stage follows infection and lasts for around 28 days on average [6]. Antibody measurement is essential for syphilis screening and diagnosis. For this reason, two types of antibodies have been employed: “non-treponemal,” which targets phospholipids, and “treponemal,” which targets T. pallidum polypeptides. The key component of syphilis control programs is antibiotic therapy, as there is no vaccination to prevent infection with T. pallidum. Parenterally administered penicillin G is suggested as the first-line treatment for incubating syphilis and for all stages of syphilis by both the UK 2008 guidelines for the management of syphilis and the U.S. Centers for Disease Control and Prevention (CDC) 2010 guidelines for the treatment of STDs [7].

This study introduces a syphilis model that incorporates partial immunity and vaccination, indicating a high likelihood of a backward bifurcation occurring in real-world syphilis dynamics [8]. Utilizing the Spectrum-STI model, another study estimates provincial trends of active syphilis among adults aged 15 to 49 in Yunnan, China, underscoring the necessity for targeted interventions to lower syphilis prevalence in high-risk groups [9]. Additionally, a comprehensive review of recent syphilis research covers clinical manifestations, diagnosis, treatment, and potential vaccines, highlighting the crucial need for biomedical interventions and alternative prevention strategies [10].

Fractional calculus is useful in a wide range of scientific domains. Researchers like different fractional operators such as AtanganaBaleanuCaputo, Fabrizio, and others have changed the fundamental definitions by substituting non-singular, nonlocal, or exponential kernels for the single kernel. The notion of generalizing classical derivative and integral definitions gave rise to fractional derivative and integral definitions [11]. The fractional order of fractional derivative definitions appears to offer many benefits over integer orders. These benefits are often evident in modeling situations that arise in the real world. In [12, 13], applications of fractional derivatives using Mittag-Leffler kernels have been studied and [14], with non-local and non-singular kernels. The kernels of the LiouvilleCaputo and RiemannLiouville definitions are solitary. This scenario results in a notable disadvantage in certain modeling scenarios. To solve this issue, Caputo-Fabrizio introduced a new definition in 2015 that had non-singularity in its kernel. Atangana-Baleanu additionally introduced a new fractional definition using Atangana-Baleanu fractional derivative in 2016 [15].

Fractional derivatives have garnered significant attention in the past few decades due to their wide-ranging applications in the mathematical modeling of dynamic systems. The memory and inheritance traits associated with fractional derivatives, along with their greater degree of freedom compared to traditional derivatives, are the primary reasons behind this surge in interest. One of the most useful tools that have been developed in recent decades to anticipate and prevent many worst-case scenarios is the ability to forecast and understand physical processes. Mathematics has undoubtedly demonstrated its usefulness in this regard, as seen by its extensive application in contemporary scientific fields including biology, physics, and epidemiology [2]. Saturated delayed impulsive effects for fractional order nonlinear systems with piecewise Caputo derivative are the subject of the work of author [16, 17]. Authors used the Mittag-Leffler kernel to analyze the HIV/AIDS model in 2022 [18]. Authors offered broad-spectrum antiviral suggestions for COVID-19 drug discovery during the COVID-19 period [19]. In 2011, scientists used a modern approach to understand the disease [20]. The severity of delayed-type hypersensitivity determines the course and symptoms of syphilis [21]. Some new results and conclusions are related to syphilis infection also studied in [22, 23]. Atangana and Baleanu [24] introduced a novel fractional integral that has been extensively researched by scholars in recent years using the RiemannLiouville fractional integral.

The authors present a novel approach to model mobile/immobile advection/dispersion in a time-fractional sense using the reproducing kernel Hilbert space method (RKHSM) and the Caputo fractional derivative [25]. In the work [26], researchers present a novel approach to model social media addiction using fractional differential equations and the reproducing kernel Hilbert space method (RKHSM). The authors present a numerical simulation of the COVID-19 pandemic using the finite element method (FEM) based on a mathematical model described by a set of ordinary differential equations [27]. The authors present a semi-analytical scheme called the Laplace Variational Iteration Method (LVIM) to solve the fractional-order blood ethanol concentration system (FBECS) [28]. The authors develop a fractional-order system to investigate the transmission dynamics of chickenpox in Phuket province of Thailand. The model includes Caputo fractional derivative and reproducing kernel Hilbert space method [29]. The paper [30], presents a novel approach to modeling the dynamics of substance addiction using fractional calculus.

The fractional piecewise operator is one of the most useful tools when studying dynamical systems including the descriptions of various diseases like SARS-COVID-19 and tuberculosis. Its importance emanates from its ability to give more precise approximations for systems that show sudden or step changes or generally exhibit discontinuities; a feature often found in real-world applications. This is a more accurate way of modeling the infection diseases by employing piecewise fractional derivatives. This more developed mathematical approach makes it possible to include memory effects and interactions that are critical in disease transmission. Furthermore, the piecewise construction of the operator enables it to mimic various interventions and changing dynamics that occur as a result of the implementation of, for example, vaccination campaigns or lockdowns. Therefore, using the fractional piecewise operator contributes to improving the ability to estimate and act on infectious diseases, leading to disease control and prevention.

Saturated delayed impulsive effects for fractional order nonlinear systems with piecewise Caputo derivatives and their applications were studied by Fei Wang [31]. For the AtanganaBaleanuCaputo model of the LIENARDS equation, Shaher Momani [32] employed convergence analysis and piecewise optimum fractional reproducing kernel solution. In reference [33], the (ABC) fractional derivative was applied to a predator-prey model involving three species. Khan et al. [34] explored the COVID-19 model using Caputo and classical piecewise operators. Atangana et al. [35] introduced a method based on piecewise differential and integral operators. Additionally, in [20], researchers studied the dynamics of a piecewise COVID-19 mathematical model incorporating vaccination and quarantine classes. This study develops a SEIR-type model for COVID-19, incorporating piecewise derivatives and stochastic differential operators. It explores the existence theory and Hyers-Ulam stability of the model and presents numerical results based on real data from Japan [36]. Another work considers a piecewise fractional-order model for COVID-19, which incorporates the impacts of the vaccines in a piecewise manner. It discusses the extinction scenario of the deterministic model and the asymptotic analysis of the solutions [37].

The purpose of using the fractional piecewise operator in the context concerns the enhancement of the modeling and analysis of the transmission of Syphilis infections. In this case, the use of this operator by different researchers is aimed at explaining the disease as complicated and potentially curable, or its ability to spread or stabilize. This paper presents a novel method of modeling syphilis based on a new line of immunity known as the fractional piecewise operator. The piecewise Atangana-Baleanu derivatives and their related numerical solution are incorporated into the model to provide better stability in terms of understanding transmission of syphilis. This new approach can be considered a major development in the manner in which the transmission rates of the syphilis illness can be determined and thus formulate a firm ground for containing the disease.

Syphilis remains a significant health problem in some regions of North America and Europe and many developing countries. Mathematical models of the dynamics of sexually transmitted infections are valuable in informing the development of control programs and in interpreting the data generated from contingent epidemiological studies. To date, their application in the research of syphilis has been restricted is the motivation of this work with the new novel hybrid fractional operator. The introduction is found in section 1 with the latest literature review. Basic definitions are covered in section 2, and the model, qualitative analysis, pose-ability, and sensitivity analysis are covered in section 3. The existence and uniqueness of a system of solutions to the ABC piecewise model is verified using the fixed point theory in section 4. We verify the Ulam-Hyers stability analysis and Lipschitz conditions in section 5. We look at the model’s numerical strategy in section 6. We go over the findings and conclusion in sections 7 and 8, respectively.

## 2 Basic concepts of fractional operator

In this section, we have some basic definitions that are useful for the analysis and study of the proposed model. Also, this will provide an overview of hybrid fractional operators, discuss their properties, and demonstrate how to numerically approximate them using well-established techniques such as Newton and Lagrange polynomials.

**Definition 1** Assume that the function F(t) is differentiable. Thus, the piecewise derivative with classical and fractional derivative with power law kernel [38, 39] is defined as follows

D0PCtβF(t)={F′(t),0<t≤t1,D0PCtβF(t),t1<t≤T, (1)

where D0Ctβ is the Caputo fractional derivative on *t*_1_ < *t* ≤ *T* and classical derivative on 0 < *t* ≤ *t*_1_.

**Definition 2** Let the function F(t) be differentiable. Thus, the piecewise integral with classical and Power law kernel [39, 40] is defined as

J0PCtβF(t)={∫0t1F(θ)dθ,0<t≤t1,1Γ(β)∫t1t(t-θ)β-1F(θ)dθ,t1<t≤T, (2)

**Definition 3** Let the function F(t) be differentiable. Thus, the piecewise derivative with classical and Mittag Leffler kernel [40] is defined as

D0PABtβF(t)={F′(t),0<t≤t1,D0PABtβF(t),t1<t≤T. (3)

**Definition 4** Let the function F(t) be differentiable. Thus, the piecewise integral with classical and Mittag Leffler kernel [40] is defined as

J0PABtβF(t)={∫0t1F(ϑ)dϑ,0<t≤t1,1-βAB(β)+βAB(β)Γ(β)∫t1t(t-ϑ)β-1F(ϑ)dϑ,t1<t≤T, (4)

where J0PABtβ stands for the mittag leffler kernel fractional integral on *t*_1_ < *t* ≤ *T* and the classical integral on 0 < *t* ≤ *t*_1_.

**Lemma 2.1** The solution of [40]:

D0PFCtϑf(t)=H(t,f(t)),

$$F\left(0\right)=F_{0}$$

is given by

$$f\left(t\right)=\{\begin{array}{c} f_{0}+\int_{0}^{t}f(\theta )d\theta ,0<t\leq t_{1},\\ f\left(t_{1}\right)+\frac{1}{\Gamma (\beta )}\int_{0}^{t}{\left(t-\theta \right)}^{\beta -1}d\theta ,t_{1}<t\leq T. \end{array}$$

## 3 Fractional order syphilis model

We adapted a mathematical model for syphilis proposed by Tiantian Zhao et al. [38]. It is a dynamic model of how syphilis spreads that we have modified to improve the model’s fit and extend its applicability when seeking to understand the transmission. The application of piecewise fractional orders plays a great role in analyzing the way models for epidemiological applications should be undertaken. This procedure flexibly estimates the parameters that allow for the real dynamics of disease spread to be estimated accurately while validating the models with real data to enhance their reliability for use in reality. In particular, by introducing piecewise fractional derivatives, scientists will be able to build models that are more resilient and adaptive, with a much deeper know-how of how infectious diseases are spreading. In so doing, this can lead to effective control strategies. Eventually, this means improving public health outcomes by providing critical insights and tools to decision-makers to fight the spread of diseases.

At a particular period t, the proposed model partitions the entire population N into four divisions. For the Syphilis disease model, we developed a fractional order mathematical version in which *S*(*t*) represents those who are at risk for infection but have not yet developed the disease, *E*(*t*) represents the number of individuals exposed, *A*(*t*) represents those who have infection, and *R*(*t*) represents those who have recovered from the illness. The first equation in our model describes how the number of the susceptible, *S*(*t*), varies over time. The equation for the number of such a conditional includes several factors that cause this reduction: contact with those infected, *σρSE*, contact with those asymptomatic, *ξτSA*, and natural mortality, *μS*. It also includes the recovery of the previously infected, *δR*, which increases the number of the susceptible. The second equation accounts for the per unit time change rate for the exposed population, *E*(*t*). It contains the terms that increase the number of exposed individuals through contact with infectives and asymptomatics, *σρSE* and *ξτSA*, and those that decrease that population through natural mortality, *μE*, pooling with the asymptomatic compartment, *ωE*, and pooling with the recovered compartment, *αE*. The third equation describes how the asymptomatic infected index *A*(*t*) changes over time. It describes how the increase in infected people who were previously exposed *ωE* and the loss of the asymptomatic pre-recovered individuals due to death *μA* and recovery *γA*. The fourth equation stands to draw the dynamics of recovered people within the population, which can be represented by *R*(*t*). It incorporates the increase in infected individuals who have recovered as they exited from the asymptomatic class *γA* and the exposed class *αE*, and the decrease in recovered individuals due to natural deaths *μR*.

{D0PCtβ(S(t))=∧-σρSE-ξτSA-μS+δR,D0PCtβ(E(t))=σρSE+ξτSA-(μ+α+w)E,D0PCtβ(A(t))=wE-(μ+d+γ)A,D0PCtβ(R(t))=γA+αE-(μ+δ)R, (5)

with the initial conditions

$$\begin{array}{c} S\left(0\right)=S_{0}>0,E\left(0\right)=E_{0}>0,A\left(0\right)=A_{0}>0,R\left(0\right)=R_{0}>0. \end{array}$$ (6)

The parameters of the model descriptions and values are given in (1).

### 3.1 Qualitative analysis of the syphilis model

To better understand the characteristics of our infectious illness model and the variables that control the dynamics of infectious disease transmission, we are now doing a qualitative analysis of the model (5).

### 3.2 Disease-free equilibrium and *R*_0_

The model’s disease-free equilibrium (DFE) is only present when there are no illnesses. Set the system’s left side to zero to solve for DFE now. As a result, the DFE of the model is obtained from system (5) by

*E*_0_ = (*S*^0^, *E*^0^, *A*^0^, *R*^0^)

$$\begin{array}{c} E_{0}=\left(\frac{\wedge }{\mu },0,0,0\right). \end{array}$$ (7)

Lets look at [38], reproductive numbers are

$$\begin{array}{c} R_{0}=\frac{\sigma \rho \Lambda }{\mu (\mu +\alpha +w)}+\frac{\xi \tau \Lambda w}{\mu (\mu +\alpha +w)(\mu +d+\gamma )}. \end{array}$$ (8)

Determining the basic reproductive number *R*_0_ of the syphilis infection model provides insight into its biological viability by quantifying its transmission potential. If *R*_0_ > 0 the infection can propagate within the population, necessitating robust intervention strategies to reduce transmission and prevent outbreaks. Understanding *R*_0_ guides public health planning, resource allocation, and the design of targeted interventions to control and manage the spread of syphilis effectively.

### 3.3 Sensitivity evaluation

In this section, we examine the sensitivity of *R*_0_ according to parameters that impact the reproduction number. The sensitivity of *R*_0_ may be investigated by taking into account given the relevant factors, partial derivatives of the reproductive number.

$$\frac{\partial R_{0}}{\partial \wedge }=\frac{\rho \sigma (\alpha +\mu +w)}{\mu }+\frac{\tau w\xi (d+\gamma +\mu )(\alpha +\mu +w)}{\mu }>0,$$

$$\frac{\partial R_{0}}{\partial \sigma }=\frac{\rho \wedge (\alpha +\mu +w)}{\mu }>0,$$

$$\frac{\partial R_{0}}{\partial \rho }=\frac{\sigma \wedge (\alpha +\mu +w)}{\mu }>0,$$

$$\frac{\partial R_{0}}{\partial \xi }=\frac{\tau w\wedge (d+\gamma +\mu )(\alpha +\mu +w)}{\mu }>0,$$

$$\frac{\partial R_{0}}{\partial \tau }=\frac{w\wedge \xi (d+\gamma +\mu )(\alpha +\mu +w)}{\mu }>0,$$

$$\frac{\partial R_{0}}{\partial \mu }=\frac{\rho \sigma \wedge }{\mu }-\frac{\rho \sigma \wedge (\alpha +\mu +w)}{\mu^{2}}+\frac{\tau w\wedge \xi (d+\gamma +\mu )}{\mu }+\frac{\tau w\wedge \xi (\alpha +\mu +w)}{\mu }-\frac{\tau w\wedge \xi (d+\gamma +\mu )(\alpha +\mu +w)}{\mu^{2}}<0,$$

$$\frac{\partial R_{0}}{\partial \alpha }=\frac{\rho \sigma \wedge }{\mu }+\frac{\tau w\wedge \xi (d+\gamma +\mu )}{\mu }>0,$$

$$\frac{\partial R_{0}}{\partial w}=\frac{\rho \sigma \wedge }{\mu }+\frac{\tau w\wedge \xi (d+\gamma +\mu )}{\mu }+\frac{\tau \wedge \xi (d+\gamma +\mu )(\alpha +\mu +w)}{\mu }>0,$$

$$\frac{\partial R_{0}}{\partial d}=\frac{\tau w\wedge \xi (\alpha +\mu +w)}{\mu }>0,$$

$$\frac{\partial R_{0}}{\partial \gamma }=\frac{\tau w\wedge \xi (\alpha +\mu +w)}{\mu }>0.$$

**Theorem 3.1** The region

$$\begin{array}{c} ℑ=\{\left(S,E,A,R\right)\in R_{+}^{4}:0<N\leq \frac{\wedge }{\mu }\}, \end{array}$$ (9)

attracts every solution in the suggested system and, in the case where the initial limitations are not negative, is positively invariant.

**Proof**: The results are provided below and shall demonstrate the system’s positive solutions.

{D0PCtβ(S(t))|S=0=∧-σρSE-ξτSA-μS+δR,≥0,D0PCtβ(E(t))|E=0=σρSE+ξτSA-(μ+α+w)E,≥0,D0PCtβ(A(t))|A=0=wE-(μ+d+γ)≥0,D0PCtβ(R(t))|R=0=γA+αE-(μ+S)R,≥0, (10)

According to the system (10), the vector field is said to be situated in the region $R_{+}^{4}$ on each hyperplane covering the non-negative orthant with *t* ≥ 0. The population’s parts from Model (5) are combined to provide the following total population.

D0PCtβN(t)=0PCDtβ(S(t))+0PCDtβ(E(t))+0PCDtβ(A(t))+0PCDtβ(R(t)), (11)

D0PCtβ(N(t))=∧-Nμ-dA.

Suppose that $N\left(0\right)\leq \frac{\wedge }{\mu }$, then we obtain that

$$\begin{array}{c} N\left(t\right)\leq \frac{\wedge }{\mu }. \end{array}$$

For any *t* > 0, a fractional model solution is thus still present in ℑ. The closed set ℑ is positively invariant concerning the fractional model. As a result, we could test our model in the hypothetical;

$$\begin{array}{c} ℑ=\{\left(S,E,A,R\right)\in R_{+}^{4}:0<N\leq \frac{\wedge }{\mu }\}. \end{array}$$

## 4 Piecewise Atangana-Baleanu syphilis model

We can express Eq (5) as

D0PCABtβS(t)={dSdt=G1(S,E,A,R,t),0<t≤t1,D0ABCtβS(t)=GABC1(S,E,A,R,t),t1<t≤T,D0PCABtβE(t)={dEdt=G2(S,E,A,R,t),0<t≤t1,D0ABtβE(t)=GABC2(S,E,A,R,t),t1<t≤T,D0PCABtβA(t)={dAdt=G3(S,E,A,R,t),0<t≤t1,D0ABCtβA(t)=GABC3(S,E,A,R,t),t1<t≤T,D0PCABtβR(t)={dRdt=G4(S,E,A,R,t),0<t≤t1,D0ABCtβR(t)=GABC4(S,E,A,R,t),t1<t≤T, (12)

To analyze the existence and uniqueness of the solution, we express the system in the following form:

$$\left\{ \begin{array}{cc} \dot{S}=G_{1}\left(S,E,A,R\right), & 0\leq t\leq W_{2},\\ \dot{E}=G_{2}\left(S,E,A,R\right), & 0\leq t\leq W_{2},\\ \dot{A}=G_{3}\left(S,E,A,R\right), & 0\leq t\leq W_{2},\\ \dot{R}=G_{4}\left(S,E,A,R\right), & 0\leq t\leq W_{2}. \end{array}\right.$$

We establish two conditions for the functions $G_{i}\left(S,E,A,R,t\right)$, where *i* = 1, 2, 3, 4, to ensure existence and uniqueness.

**Linear Growth Condition**

The functions $G_{i}$ satisfy the linear growth condition:

$$|G_{i}\left(x_{i},t\right){|}^{2}\leq K_{i}\left(1+\right|x_{i}{|}^{2}).$$

**Lipschitz Condition**

The functions $G_{i}$ satisfy the Lipschitz condition:

$$|G_{i}\left(x_{i}^{1},t\right)-G_{i}\left(x_{i}^{2},t\right){|}^{2}\leq \hat{K_{i}}{\left|x_{i}^{1}-x_{i}^{2}\right|}^{2}.$$

**Proof for Linear Growth** Consider the function $G_{1}\left(S,E,A,R\right)$:

$$G_{1}\left(S,E,A,R\right)=\wedge -\sigma \rho SE-\xi \tau SA-\mu S+\delta R.$$

We aim to show that $|G_{1}{\left(S,E,A,R\right)|}^{2}$ satisfies the linear growth condition:

$$|G_{1}{\left(S,E,A,R\right)|}^{2}\leq K_{1}{\left(1+|S\right|}^{2}).$$

First, we square $G_{1}\left(S,E,A,R\right)$:

$$|G_{1}{\left(S,E,A,R\right)|}^{2}=|\wedge -\sigma \rho SE-\xi \tau SA-\mu S+\delta R{|}^{2}.$$

Using the inequality (*a* + *b* + *c* + *d* + *e*)^2^ ≤ 5(*a*^2^ + *b*^2^ + *c*^2^ + *d*^2^ + *e*^2^), we get:

$$\begin{array}{cc} |G_{1}{\left(S,E,A,R\right)|}^{2} & \leq 5[\wedge^{2}+{\left(\sigma \rho SE\right)}^{2}+{\left(\xi \tau SA\right)}^{2}+{\left(\mu S\right)}^{2}+{\left(\delta R\right)}^{2}]\\ & =5[\wedge^{2}+\sigma^{2}\rho^{2}S^{2}E^{2}+\xi^{2}\tau^{2}S^{2}A^{2}+\mu^{2}S^{2}+\delta^{2}R^{2}]. \end{array}$$

Next, we bound each term involving *S*, *E*, *A*, and *R*:

$$\begin{array}{cc} |G_{1}{\left(S,E,A,R\right)|}^{2} & \leq 5[\wedge^{2}+\sigma^{2}\rho^{2}S^{2}{\left\|E\right\|}_{\infty }^{2}+\xi^{2}\tau^{2}S^{2}{\left\|A\right\|}_{\infty }^{2}+\mu^{2}S^{2}+\delta^{2}{\left\|R\right\|}_{\infty }^{2}]\\ & =5[\wedge^{2}+S^{2}(\sigma^{2}\rho^{2}{\left\|E\right\|}_{\infty }^{2}+\xi^{2}\tau^{2}{\left\|A\right\|}_{\infty }^{2}+\mu^{2})+\delta^{2}{\left\|R\right\|}_{\infty }^{2}]. \end{array}$$

We factor out the terms involving *S*^2^:

$$|G_{1}{\left(S,E,A,R\right)|}^{2}\leq 5[\wedge^{2}+\delta^{2}{\left\|R\right\|}_{\infty }^{2}](1+S^{2}\frac{\sigma^{2}\rho^{2}{\left\|E\right\|}_{\infty }^{2}+\xi^{2}\tau^{2}{\left\|A\right\|}_{\infty }^{2}+\mu^{2}}{\wedge^{2}+\delta^{2}{\left\|R\right\|}_{\infty }^{2}}).$$

Therefore, setting $K_{1}=5[\wedge^{2}+\delta^{2}{\left\|R\right\|}_{\infty }^{2}]$ and simplifying the fraction, we obtain:

$$|G_{1}{\left(S,E,A,R\right)|}^{2}\leq K_{1}{\left(1+|S\right|}^{2}).$$

Using similar methods, we can show that the linear growth condition holds for $G_{2}\left(S,E,A,R\right)$, $G_{3}\left(S,E,A,R\right)$, and $G_{4}\left(S,E,A,R\right)$.

$G_{2}\left(S,E,A,R\right)$

$$G_{2}\left(S,E,A,R\right)=\alpha -\beta SE-\gamma SA-\delta E+\eta R.$$

Following similar steps as above:

$$\begin{array}{cc} |G_{2}{\left(S,E,A,R\right)|}^{2} & \leq 5[\alpha^{2}+{\left(\beta SE\right)}^{2}+{\left(\gamma SA\right)}^{2}+{\left(\delta E\right)}^{2}+{\left(\eta R\right)}^{2}]\\ & =5[\alpha^{2}+\beta^{2}S^{2}{\left\|E\right\|}_{\infty }^{2}+\gamma^{2}S^{2}{\left\|A\right\|}_{\infty }^{2}+\delta^{2}{\left\|E\right\|}_{\infty }^{2}+\eta^{2}{\left\|R\right\|}_{\infty }^{2}]\\ & =5[\alpha^{2}+\delta^{2}{\left\|E\right\|}_{\infty }^{2}+\eta^{2}{\left\|R\right\|}_{\infty }^{2}+S^{2}(\beta^{2}{\left\|E\right\|}_{\infty }^{2}+\gamma^{2}{\left\|A\right\|}_{\infty }^{2})]. \end{array}$$

Setting $K_{2}=5[\alpha^{2}+\delta^{2}{\left\|E\right\|}_{\infty }^{2}+\eta^{2}{\left\|R\right\|}_{\infty }^{2}]$, we get:

$$|G_{2}{\left(S,E,A,R\right)|}^{2}\leq K_{2}{\left(1+|E\right|}^{2}).$$

$G_{3}\left(S,E,A,R\right)$

$$G_{3}\left(S,E,A,R\right)=\zeta -\kappa SE-\lambda SA-\mu A+\nu R.$$

Following similar steps as above:

$$\begin{array}{cc} |G_{3}{\left(S,E,A,R\right)|}^{2} & \leq 5[\zeta^{2}+{\left(\kappa SE\right)}^{2}+{\left(\lambda SA\right)}^{2}+{\left(\mu A\right)}^{2}+{\left(\nu R\right)}^{2}]\\ & =5[\zeta^{2}+\kappa^{2}S^{2}{\left\|E\right\|}_{\infty }^{2}+\lambda^{2}S^{2}{\left\|A\right\|}_{\infty }^{2}+\mu^{2}{\left\|A\right\|}_{\infty }^{2}+\nu^{2}{\left\|R\right\|}_{\infty }^{2}]\\ & =5[\zeta^{2}+\mu^{2}{\left\|A\right\|}_{\infty }^{2}+\nu^{2}{\left\|R\right\|}_{\infty }^{2}+S^{2}(\kappa^{2}{\left\|E\right\|}_{\infty }^{2}+\lambda^{2}{\left\|A\right\|}_{\infty }^{2})]. \end{array}$$

Setting $K_{3}=5[\zeta^{2}+\mu^{2}{\left\|A\right\|}_{\infty }^{2}+\nu^{2}{\left\|R\right\|}_{\infty }^{2}]$, we get:

$$|G_{3}{\left(S,E,A,R\right)|}^{2}\leq K_{3}{\left(1+|A\right|}^{2}).$$

$G_{4}\left(S,E,A,R\right)$

$$G_{4}\left(S,E,A,R\right)=\theta -\iota SE-\kappa SA-\lambda R+\mu E.$$

Following similar steps as above:

$$\begin{array}{cc} |G_{4}{\left(S,E,A,R\right)|}^{2} & \leq 5[\theta^{2}+{\left(\iota SE\right)}^{2}+{\left(\kappa SA\right)}^{2}+{\left(\lambda R\right)}^{2}+{\left(\mu E\right)}^{2}]\\ & =5[\theta^{2}+\iota^{2}S^{2}{\left\|E\right\|}_{\infty }^{2}+\kappa^{2}S^{2}{\left\|A\right\|}_{\infty }^{2}+\lambda^{2}{\left\|R\right\|}_{\infty }^{2}+\mu^{2}{\left\|E\right\|}_{\infty }^{2}]\\ & =5[\theta^{2}+\lambda^{2}{\left\|R\right\|}_{\infty }^{2}+\mu^{2}{\left\|E\right\|}_{\infty }^{2}+S^{2}(\iota^{2}{\left\|E\right\|}_{\infty }^{2}+\kappa^{2}{\left\|A\right\|}_{\infty }^{2})]. \end{array}$$

Setting $K_{4}=5[\theta^{2}+\lambda^{2}{\left\|R\right\|}_{\infty }^{2}+\mu^{2}{\left\|E\right\|}_{\infty }^{2}]$, we get:

$$|G_{4}{\left(S,E,A,R\right)|}^{2}\leq G_{4}{\left(1+|R\right|}^{2}).$$

Therefore, we have demonstrated that each of the functions $G_{i}\left(S,E,A,R\right)$, *i* = 1, 2, 3, 4, satisfies the linear growth condition.

**Verify the Lipschitz Condition**

To verify the Lipschitz condition for the functions $G_{i}\left(S,E,A,R\right)$, we need to show that there exist constants $\hat{G_{i}}$ such that:

$$|G_{i}\left(X^{1}\right)-{ℑ}_{i}\left(X^{2}\right)|\leq \hat{G_{i}}\left|X^{1}-X^{2}\right|,$$

where *X* denotes the relevant variable for each function.

**For** $G_{1}\left(S,E,A,R\right)$

$$G_{1}\left(S,E,A,R\right)=\wedge -\sigma \rho SE-\xi \tau SA-\mu S+\delta R.$$

We need to show:

$$|G_{1}\left(S^{1},E,A,R\right)-{ℑ}_{1}\left(S^{2},E,A,R\right)|\leq \hat{G_{1}}\left|S^{1}-S^{2}\right|.$$

Compute the difference:

$$\begin{array}{cc} |G_{1}\left(S^{1},E,A,R\right)-{ℑ}_{1}\left(S^{2},E,A,R\right)| & =|-\sigma \rho \left(S^{1}E-S^{2}E\right)-\xi \tau \left(S^{1}A-S^{2}A\right)-\mu \left(S^{1}-S^{2}\right)|\\ & =|-\sigma \rho E\left(S^{1}-S^{2}\right)-\xi \tau A\left(S^{1}-S^{2}\right)-\mu \left(S^{1}-S^{2}\right)|\\ & \leq \left|\sigma \rho E|\right|S^{1}-S^{2}\left|+|\xi \tau A|\right|S^{1}-S^{2}\left|+|\mu |\right|S^{1}-S^{2}|\\ & =(|\sigma \rho E|+|\xi \tau A|+|\mu |)\left|S^{1}-S^{2}\right|. \end{array}$$

Thus, setting $\hat{G_{1}}={\left|\sigma \rho \|E\right\|}_{\infty }{\left|+|\xi \tau \|A\right\|}_{\infty }\left|+|\mu \right|$, we obtain:

$$|G_{1}\left(S^{1},E,A,R\right)-G_{1}\left(S^{2},E,A,R\right)|\leq \hat{G_{1}}\left|S^{1}-S^{2}\right|.$$

**For** $G_{2}\left(S,E,A,R\right)$

$$G_{2}\left(S,E,A,R\right)=\alpha -\beta SE-\gamma SA-\delta E+\eta R.$$

We need to show:

$$|G_{2}\left(S,E^{1},A,R\right)-G_{2}\left(S,E^{2},A,R\right)|\leq \hat{G_{2}}\left|E^{1}-E^{2}\right|.$$

Compute the difference:

$$\begin{array}{cc} |G_{2}\left(S,E^{1},A,R\right)-{ℑ}_{2}\left(S,E^{2},A,R\right)| & =|-\beta S\left(E^{1}-E^{2}\right)-\delta \left(E^{1}-E^{2}\right)|\\ & =|\left(\beta S+\delta \right)\left(E^{1}-E^{2}\right)|\\ & \leq (|\beta S|+|\delta |)\left|E^{1}-E^{2}\right|. \end{array}$$

Thus, setting $\hat{G_{2}}={\left|\beta \|S\right\|}_{\infty }\left|+|\delta \right|$, we obtain:

$$|G_{2}\left(S,E^{1},A,R\right)-{ℑ}_{2}\left(S,E^{2},A,R\right)|\leq \hat{G_{2}}\left|E^{1}-E^{2}\right|.$$

**For** $G_{3}\left(S,E,A,R\right)$

$$G_{3}\left(S,E,A,R\right)=\zeta -\kappa SE-\lambda SA-\mu A+\nu R.$$

We need to show:

$$|G_{3}\left(S,E,A^{1},R\right)-G_{3}\left(S,E,A^{2},R\right)|\leq \hat{G_{3}}\left|A^{1}-A^{2}\right|.$$

Compute the difference:

$$\begin{array}{cc} |G_{3}\left(S,E,A^{1},R\right)-{ℑ}_{3}\left(S,E,A^{2},R\right)| & =|-\lambda S\left(A^{1}-A^{2}\right)-\mu \left(A^{1}-A^{2}\right)|\\ & =|\left(\lambda S+\mu \right)\left(A^{1}-A^{2}\right)|\\ & \leq (|\lambda S|+|\mu |)\left|A^{1}-A^{2}\right|. \end{array}$$

Thus, setting $\hat{G_{3}}={\left|\lambda \|S\right\|}_{\infty }\left|+|\mu \right|$, we obtain:

$$|G_{3}\left(S,E,A^{1},R\right)-G_{3}\left(S,E,A^{2},R\right)|\leq \hat{G_{3}}\left|A^{1}-A^{2}\right|.$$

**For** $G_{4}\left(S,E,A,R\right)$

$$G_{4}\left(S,E,A,R\right)=\theta -\iota SE-\kappa SA-\lambda R+\mu E.$$

We need to show:

$$|G_{4}\left(S,E,A,R^{1}\right)-G_{4}\left(S,E,A,R^{2}\right)|\leq \hat{G_{4}}\left|R^{1}-R^{2}\right|.$$

Compute the difference:

$$\begin{array}{cc} |G_{4}\left(S,E,A,R^{1}\right)-G_{4}\left(S,E,A,R^{2}\right)| & =|-\lambda (R^{1}-R^{2})|\\ & =|\lambda (R^{1}-R^{2})|\\ & =\left|\lambda |\right|R^{1}-R^{2}|. \end{array}$$

Thus, setting $\hat{G_{4}}=\left|\lambda \right|$, we obtain:

$$|G_{4}\left(S,E,A,R^{1}\right)-G_{4}\left(S,E,A,R^{2}\right)|\leq \hat{G_{4}}\left|R^{1}-R^{2}\right|.$$

Therefore, we have demonstrated that each of the functions $G_{i}\left(S,E,A,R\right)$, *i* = 1, 2, 3, 4, satisfies the Lipschitz condition.

**Global Solution for** [*W*_2_, *W*]

Assume {*S*(*t*), *E*(*t*), *A*(*t*), *R*(*t*)} ∈ [*W*_2_, Ψ_*e*_), where Ψ_*e*_ denotes the explosion time. We aim to show that Ψ_*e*_ = ∞.

Suppose $l_{0}\in F_{4}^{+}$ is such that $\left[\frac{1}{l_{0}},l_{0}\right]$ lies within {*S*(*W*_2_), *E*(*W*_2_), *A*(*W*_2_), *R*(*W*_2_)}. Define the stopping time:

$${\hat{\psi }}_{e}=\text{inf}\{t\geq W_{2}|\text{max}\{{\left\|S\right\|}_{\infty }{,\|E\|}_{\infty }{,\|A\|}_{\infty },{\left\|R\right\|}_{\infty }\}\geq l_{0}\}.$$

We need to show that ${\hat{\psi }}_{e}$ converges to ∞. Assume for the sake of contradiction that ${\hat{\psi }}_{e}<\infty$. This would imply that at some finite time $t={\hat{\psi }}_{e}$, one of the state variables *S*, *E*, *A*, or *R* reaches *l*_0_.

However, from the definition of ${\hat{\psi }}_{e}$, we know:

$$\text{max}\{{\left\|S\right\|}_{\infty }{,\|E\|}_{\infty }{,\|A\|}_{\infty },{\left\|R\right\|}_{\infty }\}=l_{0}\;\text{at}\;t={\hat{\psi }}_{e}.$$

Given the assumption that {*S*(*t*), *E*(*t*), *A*(*t*), *R*(*t*)} ∈ [*W*_2_, Ψ_*e*_) does not exhibit finite-time blow-up and considering the bounded nature of the system over the interval [*W*_2_, Ψ_*e*_), this leads to a contradiction.

Therefore, ${\hat{\psi }}_{e}=\infty$, which implies that the stopping time does not occur at any finite time. Hence, the solution does not blow up in finite time.

Since ${\hat{\psi }}_{e}\to \infty$, it follows that:

$$\Psi_{e}=\infty .$$

This guarantees that the solution to the system does not explode in finite time.

Thus, we have shown that the unique global solution to the system exists on the interval [0, *W*]. The system remains well-defined and bounded for all time *t* ≥ 0, ensuring that:

$$\Psi_{e}=\infty .$$

Therefore, we conclude that the unique global solution to the system exists on [0, *W*].

### 4.1 Existence and uniqueness

Next, we aim to establish the existence of a solution for the hypothetical function with piecewise derivability and investigate its particular solution properties. We can use system (12) from Lemma (2.1) to accomplish this, and for further clarification, we can also write the following:

D0PCABtβF(t)=G(t,F),0<β≤1,

is

$$\begin{array}{c} \;F\left(t\right)=\{\begin{array}{c} F_{0}+\int_{0}^{t}G(\theta ,F(\theta ))d\theta ,\;\;0<t\leq t_{1},\\ F(t_{1})+\frac{1-\beta }{ABC(\beta )}F\left(t\right)+\frac{\beta }{ABC(\beta )\Gamma (\beta )}\int_{t_{1}}^{t_{2}}{\left(t-\theta \right)}^{\beta -1}G(\theta ,F(\theta ))d\theta ,\;\;t_{1}<t\leq T, \end{array} \end{array}$$ (13)

where

$$\begin{array}{c} F=\{\begin{array}{c} S(t),\\ E(t),\\ A(t),\\ R(t), \end{array}F_{0}=\{\begin{array}{c} S_{0},\\ E_{0},\\ A_{0},\\ R_{0}, \end{array}F_{t_{1}}\{\begin{array}{c} S(t_{1}),\\ E(t_{1}),\\ A(t_{1}),\\ R(t_{1}), \end{array} \end{array}$$ (14)

G(t,F(t))={G1={GC1(S,E,A,R),GABC1(S,E,A,R),G2={GC2(S,E,A,R),GABC2(S,E,A,R),G3={GC1(S,E,A,R),GABC1(S,E,A,R),G4={GC1(S,E,A,R),GABC1(S,E,A,R), (15)

Taking ∞ > *t*_2_ ≥ *t* > *t*_1_ > 0 and the Banach space B=C[0,T] with a norm

$$\|F\|=\underset{t\in [0,T]}{\text{max}}|F(t)|.$$

We suppose that:

$$\left(A1\right)\;\;\;\;\;\;\;\;\;\exists \;L_{F}>0;\;\;\forall G,\bar{F}\in B$$

we have

$$\;\;\;\;|G\left(t,\{\right)-G\left(t,\bar{F}\right)|\leq {\text{K}}_{F}|F-\bar{F}|.$$

$$\left(A2\right)\;\;\;\;\;\;\;\;\;\exists \;C_{G}>0\;\&\;{\text{N}}_{G}>0;$$

$$\;\;\;\;|G(t,F(t))|\leq C_{G}|F|+N_{G}.$$

**Theorem 4.1** It follows that (12) has at least one solution if G be piecewise continuous on 0 < *t* ≤ *t*_1_ and *t*_1_ < *t* ≤ *T* on [0, *T*], also satisfying (*A*2).

**Proof**: Assume that closed subset B of *B* in both subintervals of [0, *T*] as

$$B=\{F\in B:\|F\|\leq P_{1,2},P>0\}.$$

Consider T:B→B and using (13) as

$$\begin{array}{c} T\left(F\right)=\{\begin{array}{c} F_{0}+\int_{0}^{t_{1}}G(\theta ,F(\theta ))d\theta ,\;\;0<t\leq t_{1},\\ F(t_{1})+\frac{1-\beta }{ABC(\beta )}F\left(t\right)+\frac{\beta }{ABC(\beta )\Gamma (\beta )}\int_{t_{1}}^{t}{\left(t-\theta \right)}^{\beta -1}G(\theta ,F(\theta ))d\theta ,\;\;t_{1}<t\leq T. \end{array} \end{array}$$

Any F∈B, we have

$$\begin{array}{c} |T\left(F\right)|\leq \{\begin{array}{c} |F_{0}|+\int_{0}^{t_{1}}|G(\theta ,F(\theta ))|d\theta ,\\ |F_{t_{1}}|+\frac{1-\beta }{ABC(\beta )}|F(t)|+\frac{\beta }{ABC(\beta )\Gamma (\beta )}\int_{t_{1}}^{t}{\left(t-\beta \right)}^{\beta -1}|G(\theta ,F(\theta ))|d\theta , \end{array}\\ \leq \{\begin{array}{c} |F_{0}|+\int_{0}^{t_{1}}\left[C_{F}|F|+N_{G}\right]d\theta ,\\ |F_{t_{1}}|+\frac{1-\beta }{ABC(\beta )}\left[C_{G}|F|+N_{G}\right]+\frac{\beta }{ABC(\beta )\Gamma (\beta )}\int_{t_{1}}^{t}{\left(t-\theta \right)}^{\beta -1}\left[C_{G}|F|+N_{G}\right]d\theta , \end{array}\\ \leq \{\begin{array}{c} |F_{0}|+t_{1}\left[C_{G}|F|+N_{G}\right]=P_{1},0<t\leq t_{1},\\ |F_{t_{1}}|+\frac{1-\beta }{ABC(\beta )}\left[C_{G}|F|+N_{G}\right]+\frac{\beta {\left(t-t_{1}\right)}^{\beta }}{ABC(\beta )\Gamma (\beta +1)}\left[C_{G}|F|+N_{G}\right]=P_{2},\;\;t_{1}<t\leq T, \end{array}\\ \leq \{\begin{array}{c} P_{1},0<t\leq t_{1},\\ P_{2},\;\;t_{1}<t\leq T. \end{array} \end{array}$$

As the prior equation establishes, as F∈B. Hence, T(B)⊂B. As a result, it proves that operator *T* is closed and completed. We can write to show further that the recommended operator is fully continuous. Using *t*_*i*_ > *t*_*j*_ ∈ [0, *t*_1_] as the initial interval for the ABC sense, consider

$$\begin{array}{ll} \left|T(F)(t_{i})-T(F)(t_{j})\right| & =|\int_{0}^{t_{i}}G\left(\theta ,F(\theta )\right)d\theta -\int_{0}^{t_{j}}F\left(\theta ,F(\theta )\right)d\theta |\\ & \leq \int_{0}^{t_{i}}\left|G\left(\theta ,F(\theta )\right)\right|d\theta -\int_{0}^{t_{j}}\left|G\left(\theta ,F(\theta )\right)\right|d\theta \\ & \leq \left[\int_{0}^{t_{i}}\left(C_{G}\left|F\right|+N_{G}\right)d\theta -\int_{0}^{t_{j}}\left(C_{G}\left|F\right|+N_{G}\right)d\theta \right]\\ & \leq \left(C_{G}\left|F\right|+N_{G}\right)\left(t_{i}-t_{j}\right). \end{array}$$ (16)

When *t*_*j*_ → *t*_*i*_, then

$$|T\left(F\right)\left(t_{i}\right)-T\left(F\right)\left(t_{j}\right)|\to 0.$$

As a result, the operator *T* equicontinuity is demonstrated in [0, *t*_1_]. Think of *t*_*i*_, *t*_*j*_ ∈ [*t*_1_, *T*] in the sense of ABC as

$$\begin{array}{ll} \left|T(F)(t_{i})-T(F)(t_{j})\right| & =|\begin{array}{l} \frac{1-\beta }{ABC(\beta )}f(t)+\frac{\beta }{ABC(\beta )\Gamma (\beta )}\int_{0}^{t_{i}}{\left(t_{i}-\theta \right)}^{\beta -1}G\left(\theta ,F(\theta )\right)d\theta \\ -\frac{1-\beta }{ABC(\beta )}F(t)+\frac{\beta }{ABC(\beta )\Gamma (\beta )}\int_{0}^{t_{j}}{\left(t_{j}-\theta \right)}^{\beta -1}G\left(\theta ,F(\theta )\right)d\theta \end{array}|\\ & \leq \frac{\beta }{ABC(\beta )\Gamma (\beta )}\int_{0}^{t_{j}}\left[{\left(t_{j}-\theta \right)}^{\beta -1}-{\left(t_{i}-\theta \right)}^{\beta -1}\right]\left|G\left(\theta ,F(\theta )\right)\right|d\theta \\ & +\frac{\beta }{ABC(\beta )\Gamma (\beta )}\int_{t_{j}}^{t_{i}}{\left(t_{i}-\theta \right)}^{\beta -1}\left|G\left(\theta ,F(\theta )\right)\right|d\theta \\ & \leq \frac{\beta }{ABC(\beta )\Gamma (\beta )}\left[\int_{0}^{t_{j}}\left[{\left(t_{j}-\theta \right)}^{\beta -1}-{\left(t_{i}-\theta \right)}^{\beta -1}\right]d\theta +\int_{t_{j}}^{t_{i}}{\left(t_{i}-\theta \right)}^{\beta -1}d\theta \right]\left(C_{G}\left|F\right|+N_{G}\right)\\ & \leq \frac{\beta \left(C_{G}F+N_{G}\right)}{ABC(\beta )\Gamma (\beta +1)}\left[t_{i}^{\beta }-t_{j}^{\beta }+2{\left(t_{i}-t_{j}\right)}^{\beta }\right]. \end{array}$$

If *t*_*j*_ → *t*_*i*_, then

$$|T\left(F\right)\left(t_{i}\right)-T\left(F\right)\left(t_{j}\right)|\to 0.$$

Thus, show that *T* is equicontinuous in [*t*_1_, *t*_2_]. As such, *T* is an equicontinuous map. The Arzela-Ascoli result demonstrates that *T* is the uniformly continuous, bounded, completely continuous operator. The Schauder result shows at least one solution for the considered model in the sub-intervals.

**Theorem 4.2** Assuming (*A*1) holds, if T is a contraction operator, the proposed piecewise model has at most one solution.

**Proof**: Since T:B→B is piecewise continuous, let F and $\bar{F}\in B$ on [0, *t*_1_] in classical form is

$$\begin{array}{cc} \left\|T\left(F\right)-T\left(\bar{F}\right)\right\| & ={\text{max}}_{t\in [0,t_{1}]}|\int_{0}^{t_{1}}G(\theta ,F(\theta ))d\theta -\int_{0}^{t_{1}}G(\theta ,\bar{F}\left(\theta \right))d\theta |\\ & \leq t_{1}L_{G}\|F-\bar{F}\|. \end{array}$$ (17)

From (17), we have

$$\begin{array}{c} \|T\left(F\right)-T\left(\bar{F}\right)\|\leq t_{1}L_{G}\|F-\bar{F}\|. \end{array}$$ (18)

*T* is, therefore, a contraction. Given the Banach result, the considered model thus has a unique solution in the given sub-interval. Moreover, *t* ∈ [*t*_1_, *t*_2_], we have

$$\begin{array}{cc} \left\|T\left(F\right)-T\left(\bar{F}\right)\right\| & =\underset{t\in [t_{1},t_{2}]}{\text{max}}|\begin{array}{c} \frac{1-\beta }{ABC(\beta )}F\left(t\right)+\frac{\beta }{ABC(\beta )\Gamma (\beta )}\int_{t_{1}}^{t_{2}}{\left(t-\theta \right)}^{\beta -1}G(\theta ,F(\theta ))d\theta \\ -\frac{1-\beta }{ABC(\beta )}\bar{F}\left(t\right)+\frac{\beta }{ABC(\beta )\Gamma (\beta )}\int_{t_{1}}^{t_{2}}{\left(t-\theta \right)}^{\beta -1}G(\theta ,\bar{F}\left(\theta \right))d\theta \end{array}|\\ & \leq \frac{1-\beta }{ABC(\beta )}L_{G}\|F-\bar{F}\|+\frac{\eta {\left(t_{2}-t_{1}\right)}^{\beta }}{ABC(\beta )\Gamma (\beta +1)}L_{G}\|F-\bar{F}\|. \end{array}$$ (19)

From (19), we have

$$\begin{array}{c} \|T\left(F\right)-T\left(\bar{F}\right)\|\leq L_{G}[\frac{1-\beta }{ABC(\beta )}+\frac{\beta {\left(t_{2}-t_{1}\right)}^{\beta }}{ABC(\beta )\Gamma (\beta +1)}]\|F-\bar{F}\|. \end{array}$$ (20)

As a result, T is a contraction. Hence, the suggested piecewise model has at most one solution.

## 5 Ulam-Hyers stability analysis

Stability is an important concern in qualitative analysis. Here, the Ulam-Hyers notion is used to establish the aforementioned component.

**Definition 5.1**

The given system of inequalities is satisfied by the solution (*S*, *E*, *A*, *R*) for every ℜ > 0, such that

|D0PCABCtβS(t)-G1(S,E,A,R)|≤ℜ,|D0PCABCtβA(t)-G2(S,E,A,R)|≤ℜ,|D0PCABCtβP(t)-G3(S,E,A,R)|≤ℜ,|D0PCABCtβR(t)-G4(S,E,A,R)|≤ℜ, (21)

if a unique solution $\left(\bar{S},\bar{E},\bar{A},\bar{R}\right)$, exists, and constant Λ > 0, it is anticipated to be Ulam-Hyers stable, holding

$$\|\left(\bar{S},\bar{E},\bar{A},\bar{R})-(S,E,A,R\right)\|\leq \Lambda ℜ.$$

Let a function h does not depend upon *S*, *E*, *A* and *R* such that *g*(0) = 0, then

**Lemma 5.1** (*i*) |*g*(*t*)| ≤ ℜ, *tε*[0, *T*],

(ii){D0PCABCtβS(t)=G1(S,E,A,R)+g(t),D0PCABCtβE(t)=G2(S,E,A,R)+g(t),D0PCABCtβA(t)=G3(S,E,A,R)+g(t),D0PCABCtβR(t)=G4(S,E,A,R)+g(t). (22)

$$S\left(0\right)=S_{0},\;E\left(0\right)=E_{0},\;A\left(0\right)=A_{0},\;R\left(0\right)=R_{0}.$$

**Lemma 5.2** The following relations are satisfied by the solution of problem (22)

$$\begin{array}{c} \begin{array}{c} |S\left(t\right)-(S\left(0\right)+\int_{0}^{t_{1}}G_{1}\left(\theta ,S,E,A,R\right)d\theta )|\leq t_{1}ℜ,t\in \left[0,t_{1}\right],\\ \;\;|S\left(t\right)-(S\left(t_{1}\right)+\frac{1-\beta }{ABC(\beta )}G_{1}+\frac{\beta }{ABC(\beta )}\int_{t_{1}}^{t}{\left(t-\theta \right)}^{\beta -1}G_{1}\left(\theta ,S,E,A,R\right)d\theta )|\\ \;\leq \left(\frac{1-\beta }{ABC(\beta )}+\frac{\beta {\left(T-\text{T}\right)}^{\beta }}{ABC(\beta )\Gamma (\beta +1)}\right)ℜ,t\in \left[t_{1},T\right], \end{array} \end{array}$$ (23)

$$\begin{array}{c} \begin{array}{c} |E\left(t\right)-(E\left(0\right)+\int_{0}^{t_{1}}G_{2}\left(\theta ,S,E,A,R\right)d\theta )|\leq t_{1}ℜ,t\in \left[0,t_{1}\right],\\ \left|E\left(t\right)-(E\left(t_{1}\right)+\frac{1-\beta }{ABC(\beta )}G_{2}+\frac{\beta }{ABC(\beta )}\int_{t_{1}}^{t}{\left(t-\theta \right)}^{\beta -1}G_{2}\left(\theta ,S,E,A,R\right)d\theta )\right|\\ \leq \left(\frac{1-\beta }{ABC(\beta )}+\frac{\beta {\left(T-\text{T}\right)}^{\beta }}{ABC(\beta )\Gamma (\beta +1)}\right)ℜ,t\in \left[t_{1},T\right], \end{array} \end{array}$$ (24)

$$\begin{array}{c} \begin{array}{c} |A\left(t\right)-(A\left(0\right)+\int_{0}^{t_{1}}G_{3}\left(\theta ,S,E,A,R\right)d\theta )|\leq t_{1}ℜ,t\in \left[0,t_{1}\right],\\ \left|A\left(t\right)-(A\left(t_{1}\right)+\frac{1-\beta }{ABC(\beta )}G_{3}+\frac{\beta }{ABC(\beta )}\int_{t_{1}}^{t}{\left(t-\theta \right)}^{\beta -1}G_{3}\left(\theta ,S,E,A,R\right)d\theta )\right|\\ \leq \left(\frac{1-\beta }{ABC(\beta )}+\frac{\beta {\left(T-\text{T}\right)}^{\beta }}{ABC(\beta )\Gamma (\beta +1)}\right)ℜ,t\in \left[t_{1},T\right], \end{array} \end{array}$$ (25)

$$\begin{array}{c} \begin{array}{c} |R\left(t\right)-(R\left(0\right)+\int_{0}^{t_{1}}G_{4}\left(\theta ,S,E,A,R\right)d\theta )|\leq t_{1}ℜ,t\in \left[0,t_{1}\right],\\ \left|R\left(t\right)-(R\left(t_{1}\right)+\frac{1-\beta }{ABC(\beta )}G_{4}+\frac{\beta }{ABC(\beta )}\int_{t_{1}}^{t}{\left(t-\theta \right)}^{\beta -1}G_{4}\left(\theta ,S,E,A,R\right)d\theta )\right|\\ \leq \left(\frac{1-\beta }{ABC(\beta )}+\frac{\beta {\left(T-\text{T}\right)}^{\beta }}{ABC(\beta )\Gamma (\beta +1)}\right)R,t\in \left[t_{1},T\right], \end{array} \end{array}$$ (26)

**Proof**: Given Lemma (2.1), a solution to Lemma (5.1) will be in the form of

$$\begin{array}{c} S\left(t\right)=\{\begin{array}{c} S_{0}+\int_{0}^{t_{1}}G_{1}\left(S,E,A,R\right)d\theta +\int_{0}^{t_{1}}g(\theta )d\theta ,t\in \left[0,t_{1}\right],\\ S\left(t_{1}\right)+\frac{1-\beta }{ABC(\beta )}G_{1}+\frac{\beta }{ABC(\beta )\Gamma (\beta )}\int_{t_{1}}^{t}{\left(t-\theta \right)}^{\beta -1}G_{1}\left(\theta ,S,E,A,R\right)d\theta \\ +\frac{1-\beta }{ABC(\beta )}g\left(t\right)+\frac{\beta }{ABC(\beta )\Gamma (\beta )}\int_{t_{1}}^{t}g(\theta )d\theta ,t\left[t_{1},T\right], \end{array} \end{array}$$ (27)

$$\begin{array}{c} E\left(t\right)=\{\begin{array}{c} E_{0}+\int_{0}^{t_{1}}G_{2}\left(S,E,A,R\right)d\theta +\int_{0}^{t_{1}}g(\theta )d\theta ,t\in \left[0,t_{1}\right],\\ E\left(t_{1}\right)+\frac{1-\beta }{ABC(\beta )}G_{2}+\frac{\beta }{ABC(\beta )\Gamma (\beta )}\int_{t_{1}}^{t}{\left(t-\theta \right)}^{\beta -1}G_{2}\left(\theta ,S,E,A,R\right)d\theta \\ +\frac{1-\beta }{ABC(\beta )}g\left(t\right)+\frac{\beta }{ABC(\beta )\Gamma (\beta )}\int_{t_{1}}^{t}g(\theta )d\theta ,t\left[t_{1},T\right], \end{array} \end{array}$$ (28)

$$\begin{array}{c} A\left(t\right)=\{\begin{array}{c} A_{0}+\int_{0}^{t_{1}}G_{3}\left(S,E,A,R\right)d\theta +\int_{0}^{t_{1}}g(\theta )d\theta ,t\in \left[0,t_{1}\right],\\ A\left(t_{1}\right)+\frac{1-\beta }{ABC(\beta )}G_{3}+\frac{\beta }{ABC(\beta )\Gamma (\beta )}\int_{t_{1}}^{t}{\left(t-\theta \right)}^{\beta -1}G_{3}\left(\theta ,S,E,A,R\right)d\theta \\ +\frac{1-\beta }{ABC(\beta )}g\left(t\right)+\frac{\beta }{ABC(\beta )\Gamma (\beta )}\int_{t_{1}}^{t}g(\theta )d\theta ,t\left[t_{1},T\right], \end{array} \end{array}$$ (29)

and

$$\begin{array}{c} R\left(t\right)=\{\begin{array}{c} R_{0}+\int_{0}^{t_{1}}G_{4}\left(S,E,A,R\right)d\theta +\int_{0}^{t_{1}}g(\theta )d\theta ,t\in \left[0,t_{1}\right],\\ R\left(t_{1}\right)+\frac{1-\beta }{ABC(\beta )}G_{4}+\frac{\beta }{ABC(\beta )\Gamma (\beta )}\int_{t_{1}}^{t}{\left(t-\theta \right)}^{\beta -1}G_{4}\left(\theta ,S,E,A,R\right)d\theta \\ +\frac{1-\beta }{ABC(\beta )}g\left(t\right)+\frac{\beta }{ABC(\beta )\Gamma (\beta )}\int_{t_{1}}^{t}g(\theta )d\theta ,t\left[t_{1},T\right], \end{array} \end{array}$$ (30)

Now, from (27)–(30) we have

$$\left\{ \begin{array}{c} |S\left(t\right)-(S_{0}+\int_{0}^{t_{1}}G_{1}\left(\theta ,S,E,A,R\right)d\theta )|\leq t_{1}ℜ,t\in \left[0,t_{1}\right],\\ \left|S\left(t\right)-(S\left(t_{1}\right)+\frac{1-\beta }{ABC(\beta )}G_{1}+\frac{\beta }{ABC(\beta )\Gamma (\beta )}\int_{t_{1}}^{t}{\left(t-\theta \right)}^{\beta -1}G_{1}\left(\theta ,S,E,A,R\right)d\theta )\right|\\ \leq \left(\frac{1-\beta }{ABC(\beta )}+\frac{\beta {\left(T-\text{T}\right)}^{\beta }}{ABC(\beta )\Gamma (\beta +1)}\right)ℜ,t\in \left[t_{1},T\right], \end{array}\right.$$

$$\left\{ \begin{array}{c} |E\left(t\right)-(E_{0}+\int_{0}^{t_{1}}G_{2}\left(\theta ,S,E,A,R\right)d\theta )|\leq t_{1}ℜ,t\in \left[0,t_{1}\right],\\ \left|E\left(t\right)-(E\left(t_{1}\right)+\frac{1-\beta }{ABC(\beta )}G_{2}+\frac{\beta }{ABC(\beta )\Gamma (\beta )}\int_{t_{1}}^{t}{\left(t-\theta \right)}^{\beta -1}G_{2}\left(\theta ,S,E,A,R\right)d\theta )\right|\\ \leq \left(\frac{1-\beta }{ABC(\beta )}+\frac{\beta {\left(T-\text{T}\right)}^{\beta }}{ABC(\beta )\Gamma (\beta +1)}\right)ℜ,t\in \left[t_{1},T\right], \end{array}\right.$$

$$\left\{ \begin{array}{c} |A\left(t\right)-(A_{0}+\int_{0}^{t_{1}}G_{3}\left(\theta ,S,E,A,R\right)d\theta )|\leq t_{1}ℜ,t\in \left[0,t_{1}\right],\\ \left|A\left(t\right)-(A\left(t_{1}\right)+\frac{1-\beta }{ABC(\beta )}G_{3}+\frac{\beta }{ABC(\beta )\Gamma (\beta )}\int_{t_{1}}^{t}{\left(t-\theta \right)}^{\beta -1}G_{3}\left(\theta ,S,E,A,R\right)d\theta )\right|\\ \leq \left(\frac{1-\beta }{ABC(\beta )}+\frac{\beta {\left(T-\text{T}\right)}^{\beta }}{ABC(\beta )\Gamma (\beta +1)}\right)ℜ,t\in \left[t_{1},T\right], \end{array}\right.$$

$$\left\{ \begin{array}{c} |R\left(t\right)-(R_{0}+\int_{0}^{t_{1}}G_{4}\left(\theta ,S,E,A,R\right)d\theta )|\leq t_{1}ℜ,t\in \left[0,t_{1}\right],\\ \left|R\left(t\right)-(R\left(t_{1}\right)+\frac{1-\beta }{ABC(\beta )}G_{4}+\frac{\beta }{ABC(\beta )\Gamma (\beta )}\int_{t_{1}}^{t}{\left(t-\theta \right)}^{\beta -1}G_{4}\left(\theta ,S,E,A,R\right)d\theta )\right|\\ \leq \left(\frac{1-\beta }{ABC(\beta )}+\frac{\beta {\left(T-\text{T}\right)}^{\beta }}{ABC(\beta )\Gamma (\beta +1)}\right)ℜ,t\in \left[t_{1},T\right], \end{array}\right.$$

**Theorem 5.1** Model (12) is Ulam-Hyers stable if *M*_1_ < 1, and *M*_2_ < 1, where

$$M_{1}=t_{1}(L_{G_{1}}+L_{G_{2}}+L_{G_{3}}+L_{G_{4}})<1$$

$$M_{2}=(\frac{1-\beta }{ABC(\beta )}+\frac{\beta {\left(T-\text{T}\right)}^{\beta }}{ABC(\beta )\Gamma (\beta +1)})(L_{G_{1}}+L_{G_{2}}+L_{G_{3}}+L_{G_{4}})<1.$$

**Proof**: If (S, E, A, R) ∈ *Y*_1_ × *Y*_2_ × *Y*_3_ × *Y*_4_ is the unique solution and $\bar{\text{S}},\bar{\text{E}},\bar{\text{A}},\bar{\text{R}},$ ∈ *Y*_1_ × *Y*_2_ × *Y*_3_ × *Y*_4_ is any solution of (12), then for *t* ∈ [0, *t*_1_], thereby in light of the Lemma (4.3) and assumption *A*_1_, one has

$$\begin{array}{c} \|S-\bar{S}\|\leq t_{1}ℜ+t_{1}L_{G_{1}}\|S-\bar{S}\|+\|E-\bar{\text{E}}\|+\|A-\bar{A}\|+\|R-\bar{R}\|, \end{array}$$ (31)

$$\begin{array}{c} \|E-\bar{E}\|\leq t_{1}ℜ+t_{1}L_{G_{2}}\|S-\bar{S}\|+\|E-\bar{\text{E}}\|+\|A-\bar{A}\|+\|R-\bar{R}\|, \end{array}$$ (32)

$$\begin{array}{c} \|A-\bar{A}\|\leq t_{1}ℜ+t_{1}L_{G_{3}}\|S-\bar{S}\|+\|E-\bar{\text{E}}\|+\|A-\bar{A}\|+\|R-\bar{R}\|, \end{array}$$ (33)

and

$$\begin{array}{c} \|R-\bar{R}\|\leq t_{1}ℜ+t_{1}L_{G_{4}}\|S-\bar{S}\|+\|E-\bar{\text{E}}\|+\|A-\bar{A}\|+\|R-\bar{R}\|, \end{array}$$ (34)

Adding (31)–(34), we have

$$\begin{array}{c} \begin{array}{c} \left\|S-\bar{S}\|+\|E-\bar{E}\|+\|A-\bar{A}\|+\|R-\bar{R}\|\leq 4t_{1}ℜ+t_{1}(L_{G_{1}}+L_{G_{2}}+L_{G_{3}}+L_{G_{4}}\right)\\ \;\times (\left\|S-\bar{S}\|+\|E-\bar{E}\|+\|A-\bar{A}\|+\|R-\bar{R}\right\|). \end{array} \end{array}$$ (35)

Let us use $M_{1}=t_{1}(L_{G_{1}}+L_{G_{2}}+L_{G_{3}}+L_{G_{4}})$, then we have from (35)

$$\begin{array}{c} \|\left(A,E,A,R)-(\bar{S},E,\bar{A},\bar{R}\right)\|\leq \frac{4t_{1}}{1-M_{1}}ℜ,t\in [0,t_{1}]. \end{array}$$ (36)

For *t* ∈ (*t*_1_, *T*], we have

$$\begin{array}{c} \begin{array}{c} \left\|S-\bar{S}\|\leq (\frac{1-\beta }{ABC(\beta )}+\frac{\beta {\left(T-\text{T}\right)}^{\beta }}{ABC(\beta )\Gamma (\beta +1)})ℜ+(\frac{1-\beta }{ABC(\beta )}+\frac{\beta {\left(T-\text{T}\right)}^{\beta }}{ABC(\beta )\Gamma (\beta +1)}\right)\\ \;\times (\left\|S-\bar{S}\|+\|E-\bar{E}\|+\|A-\bar{A}\|+\|R-\bar{R}\right\|), \end{array} \end{array}$$ (37)

$$\begin{array}{c} \begin{array}{c} \left\|E-\bar{E}\|\leq (\frac{1-\beta }{ABC(\beta )}+\frac{\beta {\left(T-\text{T}\right)}^{\beta }}{ABC(\beta )\Gamma (\beta +1)})ℜ+(\frac{1-\beta }{ABC(\beta )}+\frac{\beta {\left(T-\text{T}\right)}^{\beta }}{ABC(\beta )\Gamma (\beta +1)}\right)\\ \;\times (\left\|S-\bar{S}\|+\|E-\bar{E}\|+\|A-\bar{A}\|+\|R-\bar{R}\right\|), \end{array} \end{array}$$ (38)

$$\begin{array}{c} \begin{array}{c} \left\|A-\bar{A}\|\leq (\frac{1-\beta }{ABC(\beta )}+\frac{\beta {\left(T-\text{T}\right)}^{\beta }}{ABC(\beta )\Gamma (\beta +1)})ℜ+(\frac{1-\beta }{ABC(\beta )}+\frac{\beta {\left(T-\text{T}\right)}^{\beta }}{ABC(\beta )\Gamma (\beta +1)}\right)\\ \;\times (\left\|S-\bar{S}\|+\|E-\bar{E}\|+\|A-\bar{A}\|+\|R-\bar{R}\right\|), \end{array} \end{array}$$ (39)

$$\begin{array}{c} \begin{array}{c} \left\|R-\bar{R}\|\leq (\frac{1-\beta }{ABC(\beta )}+\frac{\beta {\left(T-\text{T}\right)}^{\beta }}{ABC(\beta )\Gamma (\beta +1)})ℜ+(\frac{1-\beta }{ABC(\beta )}+\frac{\beta {\left(T-\text{T}\right)}^{\beta }}{ABC(\beta )\Gamma (\beta +1)}\right)\\ \;\times (\left\|S-\bar{S}\|+\|E-\bar{E}\|+\|A-\bar{A}\|+\|R-\bar{R}\right\|), \end{array} \end{array}$$ (40)

Adding (37)–(40), we obtain

$$\begin{array}{c} \begin{array}{c} \begin{array}{c} \|\left(S,E,A,R,)-(\bar{S},\bar{E},\bar{A},\bar{R}\right)\|\leq 4(\frac{1-\beta }{ABC(\beta )}+\frac{\beta {\left(T-\text{T}\right)}^{\beta }}{ABC(\beta )\Gamma (\beta +1)})ℜ\\ \;+(\frac{1-\beta }{ABC(\beta )}+\frac{\beta {\left(T-\text{T}\right)}^{\beta }}{ABC(\beta )\Gamma (\beta +1)})(L_{G_{1}}+L_{G_{2}}+L_{G_{3}}+L_{G_{4}}) \end{array}\\ \;\times \|\left(A,E,A,R)-(\bar{A},\bar{E},\bar{A},\bar{R}\right)\|. \end{array} \end{array}$$ (41)

For easiness, we put $M_{2}=(\frac{1-\beta }{ABC(\beta )}+\frac{\beta {\left(T-\text{T}\right)}^{\beta }}{ABC(\beta )\Gamma (\beta +1)})(L_{G_{1}}+L_{G_{2}}+L_{G_{3}}+L_{G_{4}}),M_{3}=4(\frac{1-\beta }{ABC(\beta )}+\frac{\beta {\left(T-\text{T}\right)}^{\beta }}{ABC(\beta )\Gamma (\beta +1)}),$ then (41) yield

$$\begin{array}{c} \|\left(S,E,A,R)-(\bar{S},E,\bar{A},\bar{R}\right)\|\leq \frac{M_{3}}{1-M_{2}}ℜ,t\in [t_{1},T]. \end{array}$$ (42)

Since *M*_1_ < 1, and *M*_2_ < 1. Therefore, 1 − *M*_1_ ≠ 0, 1 − *M*_2_ ≠ 0, hence from (35) and (41) with $\text{max}\{\frac{4t_{1}}{1-M_{1}},\frac{M_{3}}{1-M_{2}}\}=\Delta ,$ one has

$$\begin{array}{c} \|\left(S,E,A,R)-(\bar{S},\bar{E},\bar{A},\bar{R}\right)\|\leq \Delta ℜ. \end{array}$$ (43)

Ulam-Hyers stability results in the solution. Additionally, whenever a function Φ(constant or rising) from *R*^+^, *R*^+^ exists such that Φ(ℜ) = ℜ. Using (43), we have the capability to get

$$\begin{array}{c} \|\left(S,E,A,R)-(\bar{S},\bar{E},\bar{A},\bar{R}\right)\|\leq \Delta \;\Phi (ℜ), \end{array}$$ (44)

with Φ(0) = 0, which proves that considered system is generalized Ulam-Hyers stable.

**Remarks 1** The conditions for Ulam-Hyer’s stability in the syphilis transmission model can be practically ensured by appropriately selecting and adjusting model parameters. Specifically, the stability condition $M_{1}=t_{1}(L_{G_{1}}+L_{G_{2}}+L_{G_{3}}+L_{G_{4}})<1$ is satisfied by choosing a suitable time interval *t*_1_, while $M_{2}=(\frac{1-\beta }{ABC(\beta )}+\frac{\beta {\left(T-\text{T}\right)}^{\beta }}{ABC(\beta )\Gamma (\beta +1)})(L_{G_{1}}+L_{G_{2}}+L_{G_{3}}+L_{G_{4}})<1$ is achieved through careful management of the fractional terms and the time variables *T* and **T**.

**Remarks 2** The Generalized Ulam-Hyers stability results ensure global stability in the investigated model by rigorously analyzing its response to perturbations. This increases confidence in the proposed mathematical framework in evaluating the interventions and the decision-making within the syphilis management as it gives a strong foundation of the dependency of solutions on some constants. These results are informative in the competitive environment concerning the efficiency of intervention strategies and their vulnerability in real-world conditions, which is important for strategic decision-making.

## 6 Numerical scheme

This Newton polynomial piecewise fractional approach is very useful in the modeling and numerical simulation of systems which at some particular instance experiences sudden changes or else are in the switch- like condition. It is appropriate for analyzing the real-world phenomena due to the reasons of spaciousness, speed in computations and identification of various patterns of system behavior in infectious diseases. As per the work of Atangana [40], the authors have applied a generalized scheme which has nonlocal and nonsingular kernel characteristics and the authors have proved it first on the first equation of *t*_*ℓ*+1_ and then on the above mentioned equations.

$$\begin{array}{c} \text{S}\left(t\right)=\{\begin{array}{c} S_{0}+\int_{0}^{t_{1}}G_{1}\left(\theta ,S,E,A,R\right)d\theta ,0<t\leq t_{1},\\ S\left(t_{1}\right)+\frac{1-\beta }{AB(\beta )}G_{1}\left(t,S,E,A,R\right)\\ +\frac{\beta }{ABC(\beta )\Gamma (\beta )}\int_{t_{1}}^{t_{2}}{\left(t-\theta \right)}^{\beta -1}G_{1}\left(\theta ,S,E,A,R\right)d\theta ,t_{1}<t\leq t_{2}, \end{array} \end{array}$$ (45)

$$\begin{array}{c} S\left(t_{\ell +1}\right)=\{\begin{array}{c} S_{0}+\sum_{†=2}^{j}[\frac{5}{12}G_{1}\left(H_{2},t_{m-2}\right)\Lambda t-\\ \frac{4}{3}G_{1}\left(H_{1},t_{m-1}\right)\Lambda t+G_{1}\left(H,t_{\kappa }\right)],\\ S\left(t_{1}\right)+\frac{1-\beta }{AB(\beta )}G_{1}\left(S^{\ell },E^{\ell },A^{\ell },R^{\ell }\right)+\\ \frac{\beta }{AB(\beta )}\frac{{\left(\Lambda t\right)}^{\beta -1}}{ℵ(\beta +1)}\sum_{†=\upsilon +3}^{\ell }\left[G_{1}\left(H_{2},t_{†-2}\right)\right]\gamma +\\ \frac{\beta }{AB(\beta )}\frac{{\left(\Lambda t\right)}^{\ell -1}}{ℵ(\beta +2)}\sum_{†=\upsilon +3}^{\ell }\left[G_{1}\left(H_{2},t_{†-1}\right)-G_{1}\left(H_{2},t_{†-2}\right)\right]\phi +\\ \frac{\beta }{AB(\beta )}\frac{{\left(\Lambda t\right)}^{\ell -1}}{2ℵ(\beta +3)}\sum_{†=\upsilon +3}^{\ell }[G_{1}\left(H,t_{†}\right)-2G_{1}\left(H_{1},t_{†-1}\right)+G_{1}\left(H_{2},t_{†-2}\right]\Lambda \end{array} \end{array}$$ (46)

$$\begin{array}{c} E\left(t_{\ell +1}\right)=\{\begin{array}{c} E_{0}+\sum_{†=2}^{j}[\frac{5}{12}G_{2}\left(H_{2},t_{m-2}\right)\Lambda t-\\ \frac{4}{3}G_{2}\left(H_{1},t_{m-1}\right)\Lambda t+G_{2}\left(H,t_{\kappa }\right)],\\ E\left(t_{1}\right)+\frac{1-\beta }{AB(\beta )}G_{2}\left(S^{\ell },E^{\ell },A^{\ell },R^{\ell }\right)+\\ \frac{\beta }{AB(\beta )}\frac{{\left(\Lambda t\right)}^{\beta -1}}{ℵ(\beta +1)}\sum_{†=\upsilon +3}^{\ell }\left[G_{2}\left(H_{2},t_{†-2}\right)\right]\gamma +\\ \frac{\beta }{AB(\beta )}\frac{{\left(\Lambda t\right)}^{\ell -1}}{ℵ(\beta +2)}\sum_{†=\upsilon +3}^{\ell }\left[G_{2}\left(H_{2},t_{†-1}\right)-G_{2}\left(H_{2},t_{†-2}\right)\right]\phi +\\ \frac{\beta }{AB(\beta )}\frac{{\left(\Lambda t\right)}^{\ell -1}}{2ℵ(\beta +3)}\sum_{†=\upsilon +3}^{\ell }[G_{2}\left(H,t_{†}\right)-2G_{2}\left(H_{1},t_{†-1}\right)+G_{2}\left(H_{2},t_{†-2}\right]\Lambda \end{array} \end{array}$$ (47)

$$\begin{array}{c} A\left(t_{\ell +1}\right)=\{\begin{array}{c} A_{0}+\sum_{†=2}^{j}[\frac{5}{12}G_{3}\left(H_{2},t_{m-2}\right)\Lambda t-\\ \frac{4}{3}G_{3}\left(H_{1},t_{m-1}\right)\Lambda t+G_{3}\left(H,t_{\kappa }\right)],\\ A\left(t_{1}\right)+\frac{1-\beta }{AB(\beta )}G_{3}\left(S^{\ell },E^{\ell },A^{\ell },R^{\ell }\right)+\\ \frac{\beta }{AB(\beta )}\frac{{\left(\Lambda t\right)}^{\beta -1}}{ℵ(\beta +1)}\sum_{†=\upsilon +3}^{\ell }\left[G_{3}\left(H_{2},t_{†-2}\right)\right]\gamma +\\ \frac{\beta }{AB(\beta )}\frac{{\left(\Lambda t\right)}^{\ell -1}}{ℵ(\beta +2)}\sum_{†=\upsilon +3}^{\ell }\left[G_{3}\left(H_{2},t_{†-1}\right)-G_{3}\left(H_{2},t_{†-2}\right)\right]\phi +\\ \frac{\beta }{AB(\beta )}\frac{{\left(\Lambda t\right)}^{\ell -1}}{2ℵ(\beta +3)}\sum_{†=\upsilon +3}^{\ell }[G_{3}\left(H,t_{†}\right)-2G_{3}\left(H_{1},t_{†-1}\right)+G_{3}\left(H_{2},t_{†-2}\right]\Lambda \end{array} \end{array}$$ (48)

$$\begin{array}{c} R\left(t_{\ell +1}\right)=\{\begin{array}{c} R_{0}+\sum_{†=2}^{j}[\frac{5}{12}G_{4}\left(H_{2},t_{m-2}\right)\Lambda t-\\ \frac{4}{3}G_{4}\left(H_{1},t_{m-1}\right)\Lambda t+G_{4}\left(H,t_{\kappa }\right)],\\ R\left(t_{1}\right)+\frac{1-\beta }{AB(\beta )}G_{4}\left(S^{\ell },E^{\ell },A^{\ell },R^{\ell }\right)+\\ \frac{\beta }{AB(\beta )}\frac{{\left(\Lambda t\right)}^{\beta -1}}{ℵ(\beta +1)}\sum_{†=\upsilon +3}^{\ell }\left[G_{4}\left(H_{2},t_{†-2}\right)\right]\gamma +\\ \frac{\beta }{AB(\beta )}\frac{{\left(\Lambda t\right)}^{\ell -1}}{ℵ(\beta +2)}\sum_{†=\upsilon +3}^{\ell }\left[G_{4}\left(H_{2},t_{†-1}\right)-G_{4}\left(H_{2},t_{†-2}\right)\right]\phi +\\ \frac{\beta }{AB(\beta )}\frac{{\left(\Lambda t\right)}^{\ell -1}}{2ℵ(\beta +3)}\sum_{†=\upsilon +3}^{\ell }[G_{4}\left(H,t_{†}\right)-2G_{4}\left(H_{1},t_{†-1}\right)+G_{4}\left(H_{2},t_{†-2}\right]\Lambda \end{array} \end{array}$$ (49)

where

$$H=S^{†},E^{†},A^{†},R^{†}$$

$$H_{1}=S^{†-1},E^{†-1},A^{†-1},R^{†-1}$$

$$H_{2}=S^{†-2},E^{†-2},A^{†-2},R^{†-2}$$

and

$$\Lambda =[{\left(1+\ell -†\right)}^{\beta }\left(2{\left(\ell -†\right)}^{2}+\left(3\beta +10\right)\left(\ell -†\right)+2\beta^{2}+9\beta +12\right)-$$

$$\left(\ell -†\right)(2{\left(\ell -†\right)}^{2}+\left(5\beta +10\right)\left(\ell -†\right)+6\beta^{2}+18\beta +12)],$$

$$\gamma =[{\left(1+\ell -†\right)}^{\beta }\left(3+2\beta -†+\ell \right)-\left(\ell -†\right)\left(3+3\beta -†+\ell \right)],$$

$$\phi =[{\left(1+\ell -†\right)}^{\beta }-{\left(\ell -†\right)}^{\beta }]$$

## 7 Results of proposed scheme

**Fig 1:**
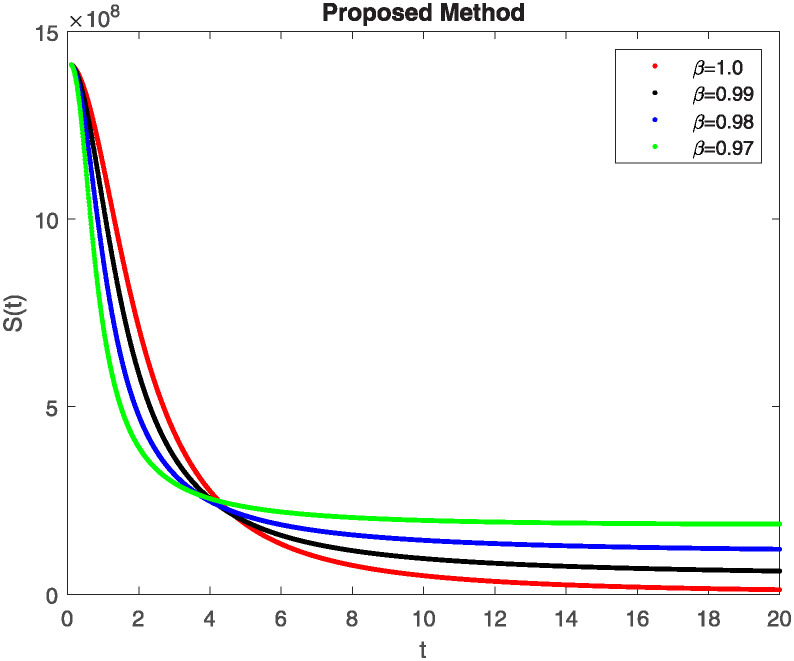
The dynamics of S(t) at diffident fractional order values with piecewise ABC.

**Fig 2:**
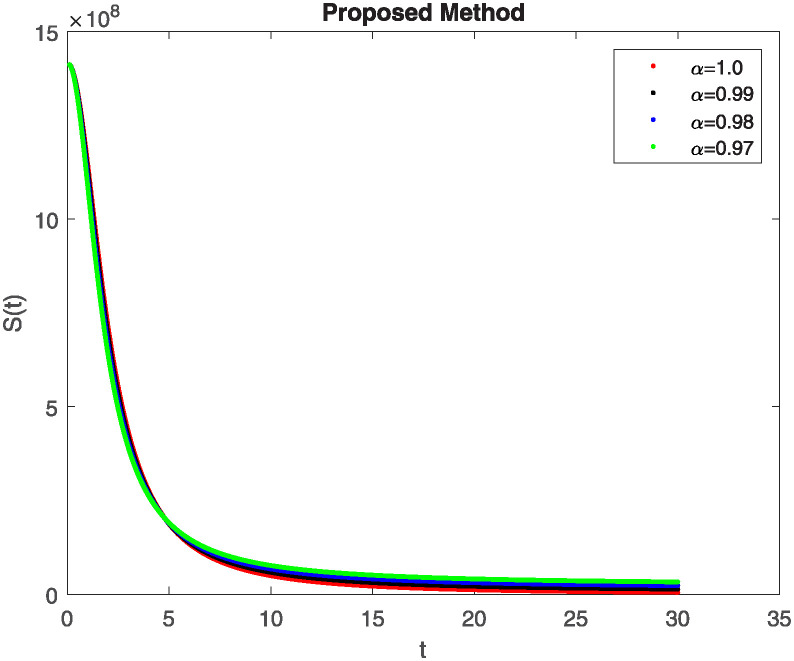
The dynamics of S(t) at diffident fractional order values with piecewise caputo.

**Fig 3:**
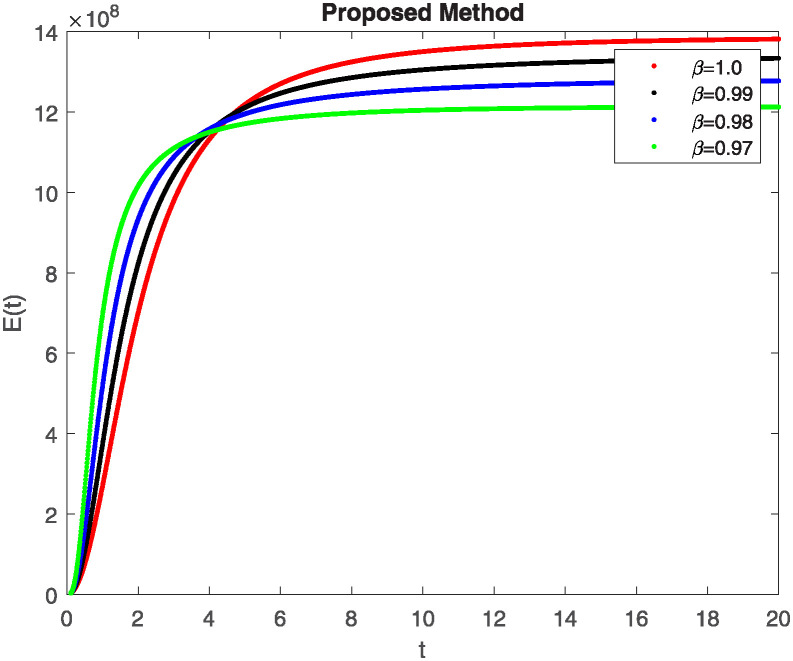
The dynamics of E(t) at diffident fractional order values with piecewise ABC.

**Fig 4:**
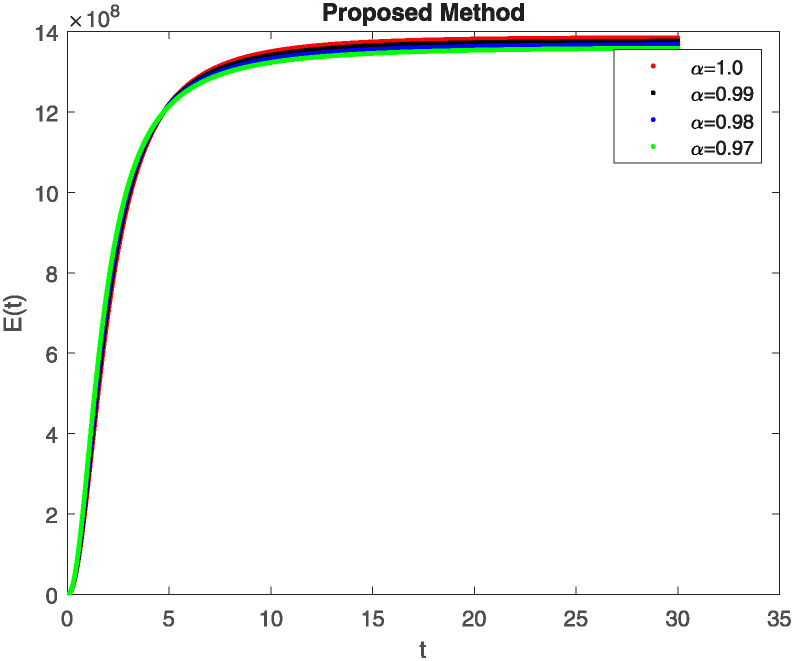
The dynamics of E(t) at diffident fractional order values with piecewise caputo.

**Fig 5:**
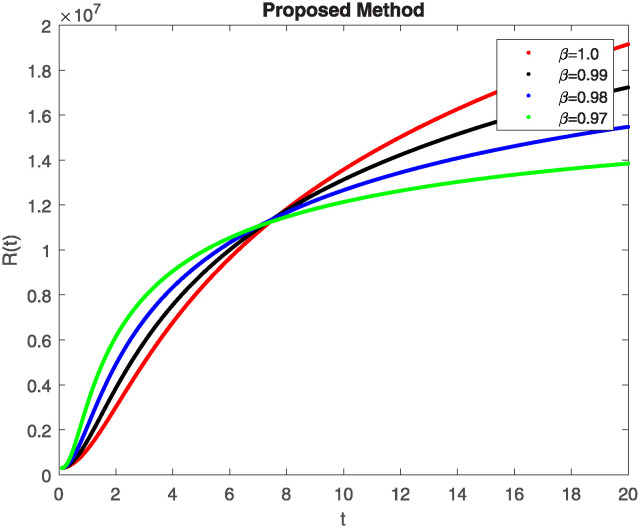
The dynamics of A(t) at diffident fractional order values with piecewise ABC.

**Fig 6:**
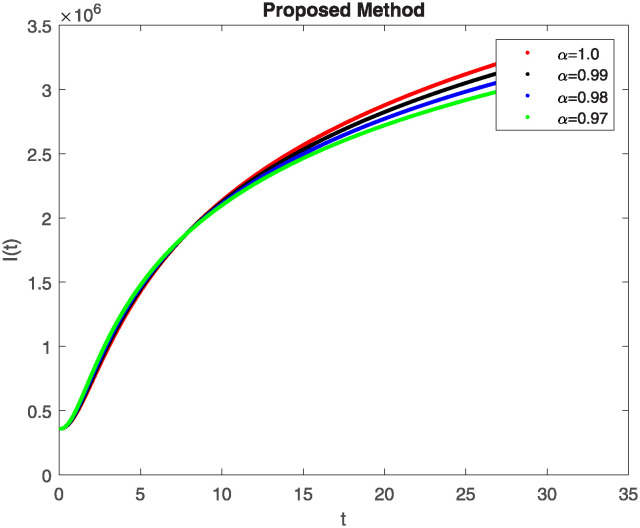
The dynamics of A(t) at diffident fractional order values with piecewise caputo.

**Fig 7:**
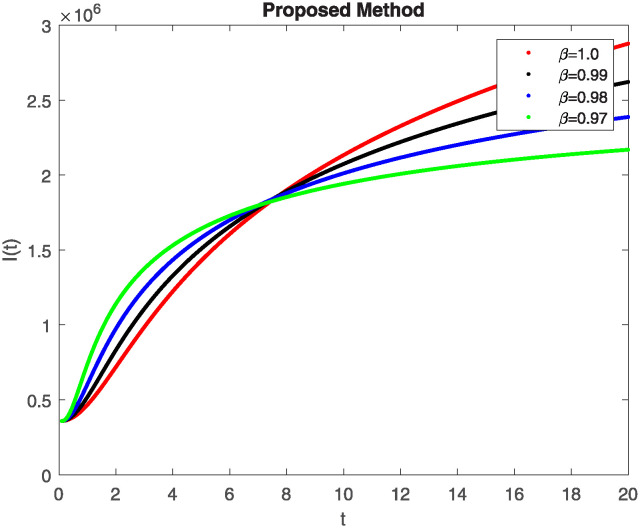
The dynamics of R(t) at diffident fractional order values with piecewise ABC.

**Fig 8:**
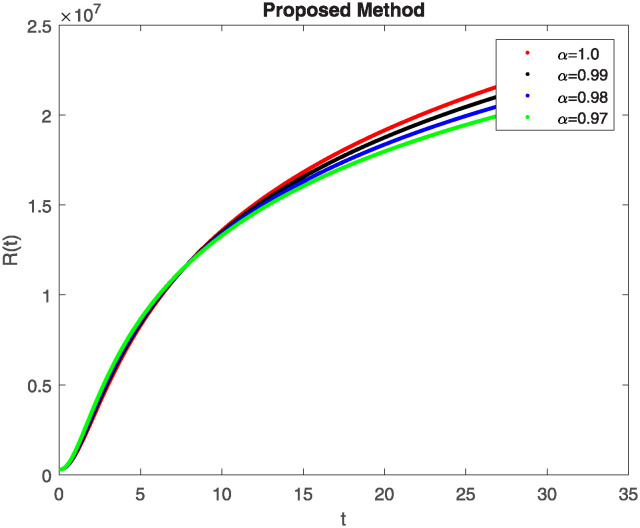
The dynamics of R(t) at diffident fractional order values with piecewise caputo.

**Fig 9:**
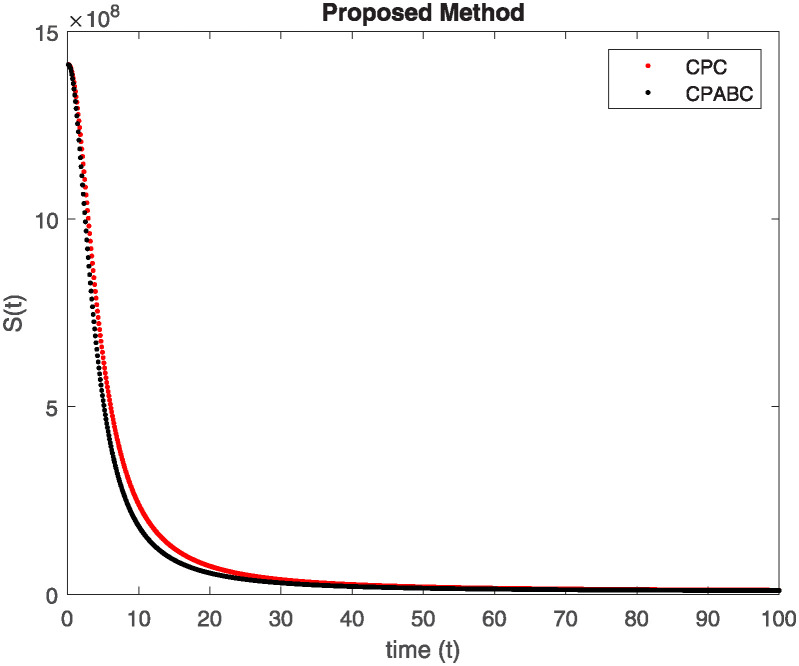
Comparison of classicalpiecewise AB Caputo and Caputo derivative at fractional order 0.99 for S(t).

**Fig 10:**
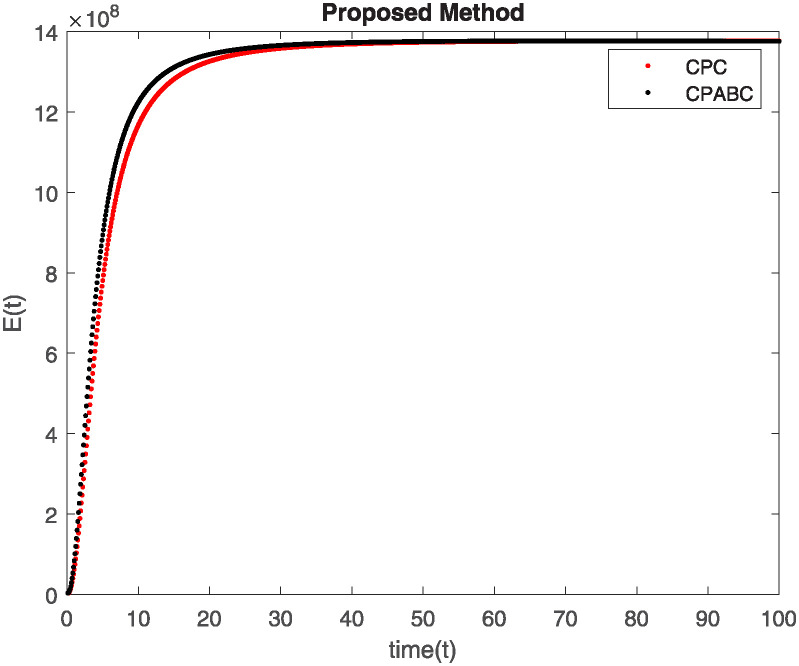
Comparison of classicalpiecewise AB Caputo and Caputo derivative at fractional order 0.99 for E(t).

**Fig 11:**
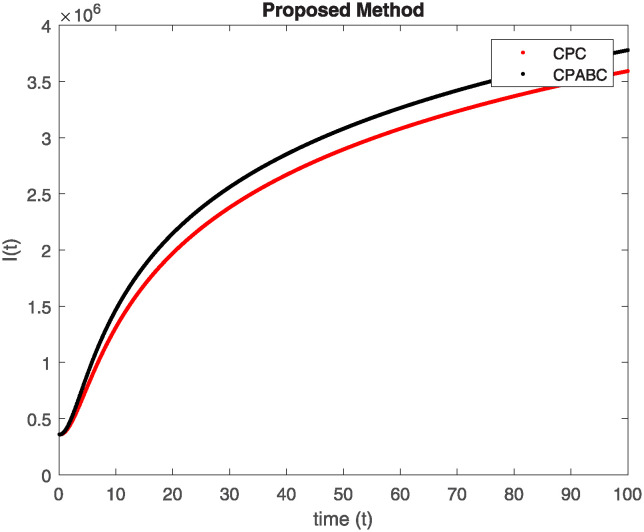
Comparison of classicalpiecewise AB Caputo and Caputo derivative at fractional order 0.99 for A(t).

**Fig 12:**
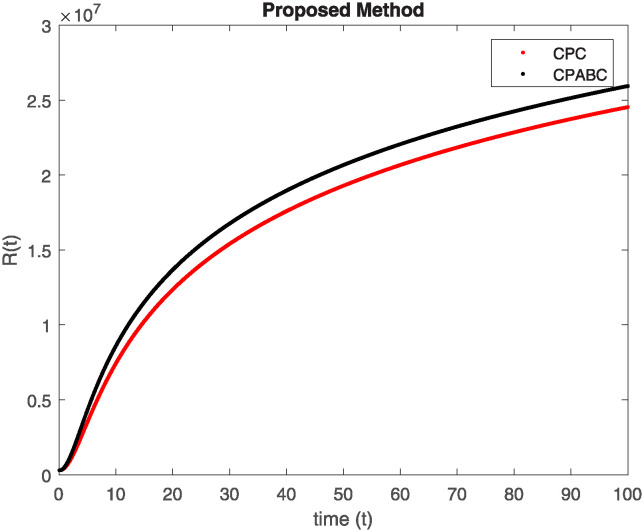
Comparison of classicalpiecewise AB Caputo and Caputo derivative at fractional order 0.99 for R(t).

**Fig 13:**
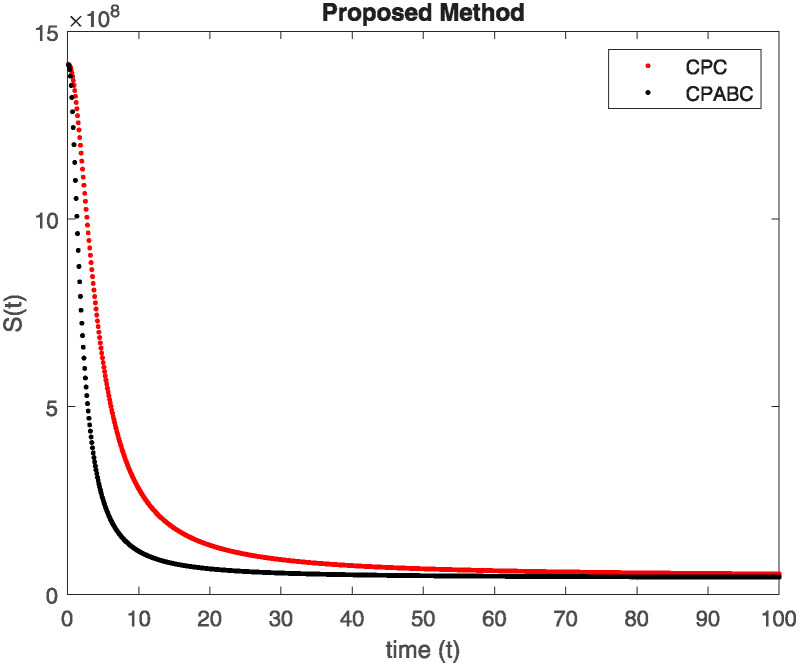
Comparison of classicalpiecewise AB Caputo and Caputo derivative at fractional order 0.9 for S(t).

**Fig 14:**
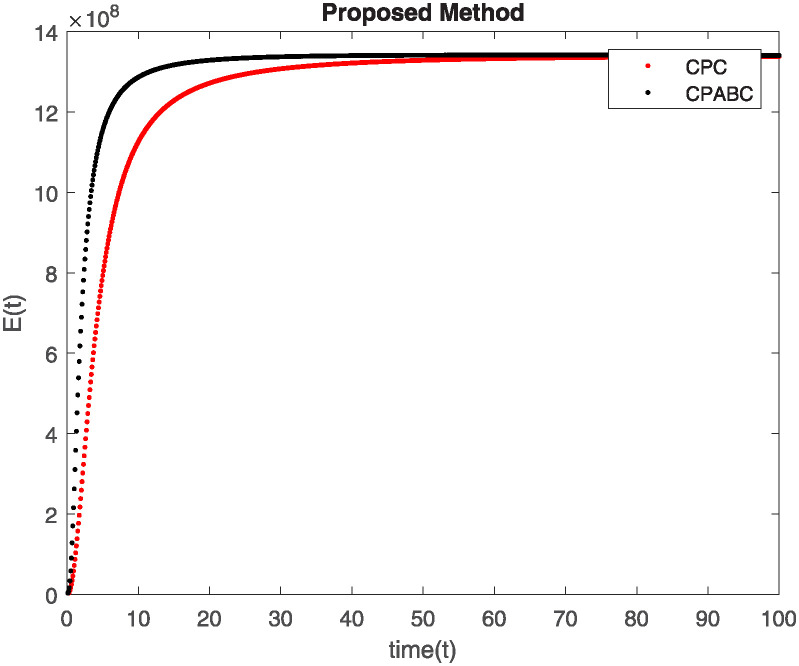
Comparison of classicalpiecewise AB Caputo and Caputo derivative at fractional order 0.9 for E(t).

**Fig 15:**
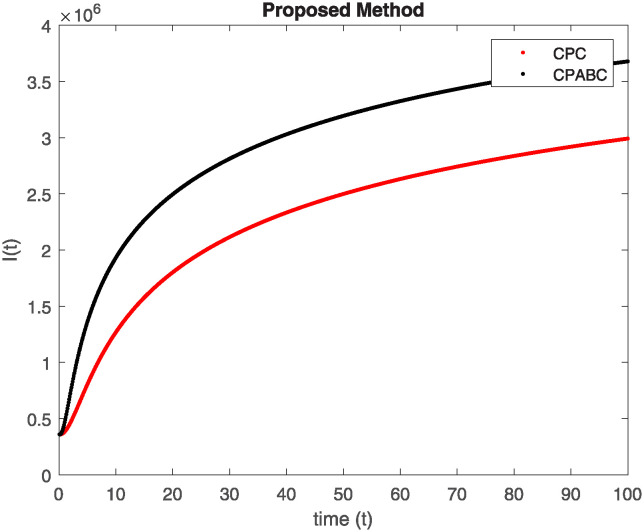
Comparison of classicalpiecewise AB Caputo and Caputo derivative at fractional order 0.9 for A(t).

**Fig 16:**
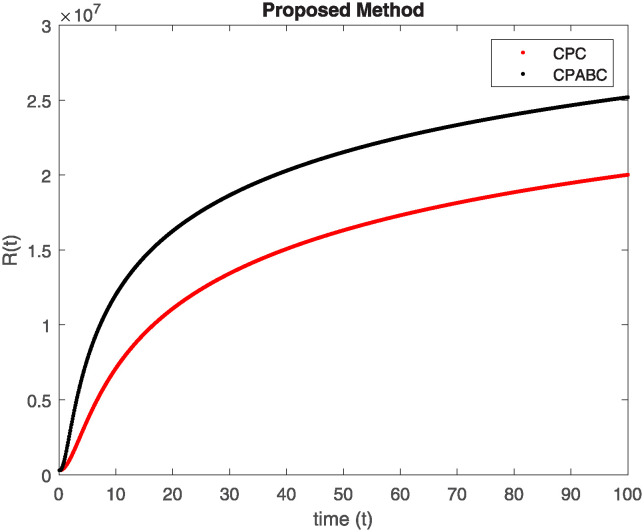
Comparison of classicalpiecewise AB Caputo and Caputo derivative at fractional order 0.9 for R(t).

We conducted a mathematical investigation into syphilis, specifically exploring its behavior using fractional operators to understand its real impact. The initial values *S*(0) = 1411780000, *E*(0) = 3000000, *A*(0) = 358534, *R*(0) = 295000 and parameters for systems (5) are listed in Table 1 and were taken from [38]. Unexpected reactions resulting from the sections of the postulated fractional order model are obtained by utilizing non-integer values of parameters of the proposed operator as shown in Figs 1–16. Simulations are drawn both with piecewise ABC and Caputo is also shown in figures. Fig 1 shows the dynamics of S(t) using the piecewise ABC derivative, and Fig 2 shows the dynamics of S(t) using the piecewise Caputo derivative. The curves in both subplots represent different fractional orders (*β*) with values 1.0, 0.99, 0.98, and 0.97. In general, S(t), representing the susceptible population, decreases over time in both sub-figures, indicating a reduction in the number of susceptible individuals as time progresses. When comparing the piecewise ABC and Caputo methods, both display a similar overall decreasing trend for S(t). Although the rate of decrease is slightly different between the methods, the overall behavior remains consistent, with a continuous decline in the susceptible population. Fig 3 illustrates the dynamics of E(t)(exposed population) under different fractional order values. Fig 3 depicts E(t) with the Piecewise ABC method, while Fig 4 shows E(t) with the Piecewise Caputo method. The curves in both subplots represent different fractional orders (*β*) of 1.0, 0.99, 0.98, and 0.97. Generally, E(t) increases initially and then decreases over time in both methods, indicating a rise to a peak followed by a decline in the exposed population. While both methods show this initial rise and subsequent fall, the timing and peak of E(t) vary slightly between them. Fig 5 illustrates the dynamics of *A*(*t*) using two operators, piecewise ABC and piecewise Caputo, with different fractional orders (*β*) of 1.0, 0.99, 0.98, and 0.97. Fig 5 depicts *A*(*t*) using the piecewise ABC operator, while Fig 6 shows *A*(*t*) using the piecewise Caputo oprator. In both subplots, *A*(*t*) rises to a peak before declining, indicating that the infected population increases to a maximum point and then decreases over time. Both methods show this trend, but the peak position and height differ slightly between them, with variations in the exact timing and maximum number of infected individuals. Fig 7 shows the dynamics of R(t) using the piecewise ABC derivative, and Fig 8 shows the dynamics of R(t) using the piecewise Caputo derivative. The curves in both subplots represent different fractional orders (*β*) with values 1.0, 0.99, 0.98, and 0.97. In general, R(t), representing the recovered population, increases over time in both sub-figures. This indicates a continuous growth in the number of individuals recovering from the infection. There are minor differences in the growth rate between the operators. Although the overall trajectory remains similar, slight variations in the rate of increase can be observed, indicating nuanced differences in the dynamics of recovery between the piecewise ABC and Caputo approaches.

**Table 1:** Model parameters and their sources.

Parameter	Description	Value	Source
Λ	Recruitment rate of individuals	10,010,000	[38]
*μ*	Natural death rate	0.00709	[38]
*d*	Death rate due to disease	0.0001393	[38]
*c*_1_	Rate of sexual partner change during incubation	20	[38]
*c*_2_	Rate of sexual partner change during infection	20	[38]
*β*_1_	Transmission probability during incubation	0.1211 × 10^−7^	[38]
*β*_2_	Transmission probability during infection	0.2691 × 10^−7^	[38]
*w*	Rate of progression from incubation to infection	0.13	[38]
*γ*	Treatment rate during infection stage	0.8	[38]
*α*	Treatment rate during incubation stage	0.9626	[38]
*δ*	Relapse rate of recovered individuals returning to susceptible status	1.6590 × 10^−6^	[38]

Figs 9–14 provide a comparative analysis between the classical Piecewise AB Caputo and Caputo derivative methods across fractional orders 0.99, 0.95, and 0.9. The differences between the Piecewise AB Caputo and Caputo derivative methods are subtle, but they are nonetheless discernible. Specifically, variations in the timing and magnitude of peaks and troughs become apparent when comparing the output of the two methods. These nuances highlight the distinct computational strategies employed by each method and their respective impacts on the modeled dynamics. Such differences may have implications for the interpretation and prediction of system behavior, particularly in scenarios where precise timing or magnitude of events is crucial. In Figs 15 and 16, the dynamics of the system at a fractional order of 0.8 are compared between the Caputo and ABC methods. This fractional order represents a lower value compared to the previous comparisons, leading to slower dynamics in both methods when compared to higher fractional orders. The other difference that can be observed between the Caputo and ABC methods is in the degree of smoothness of the transitions. Namely, the use of the ABC method implies a smoother transition between various phases of the epidemic as compared to the usage of the Caputo method. From this, it can be logically deduced that with the ABC method, the changes in the variables such as S(t), E(t), A(t), and R(t) appear to be more gradual. At the same time, the Caputo method can lead to greater changes at particular moments and, therefore, make the transitions between the phases of the epidemic clearer.

The results obtained with the proposed operator demonstrate enhanced conformity, as they closely correspond with the steady-state equilibrium points across various fractional order values. Memory influence can be observed with a greater degree of freedom and a solution restricted to a steady state point that lies in a feasible range by varying the fractional order derivative. Our comparison between the Atangana-Baleanu Caputo derivative and classical operators like the traditional Caputo operator offers valuable insights into the efficiency and accuracy of numerical approaches in modeling syphilis infection dynamics. By evaluating computational costs and complexities, we have elucidated their efficiency in capturing the intricate disease transmission dynamics. The task of evaluating accuracy of real-world outcome prediction and identifying differences between numerical methods has become an essential issue in the choice of the proper numerical method. This analysis not only helps in the identification of methodological development but also provides insights for generating more accurate and efficient numeric techniques for the exploration of dynamics of syphilis infections and similar systems, providing the progress the studies of infectious disease and the planning in public health.

## 8 Conclusion

In this work, we employed the piecewise operator in the context of the classical ABC Caputo sense to assess the characteristics of the syphilis model. The equilibrium points and locally global stability of a solution including a piecewise ABC derivative are examined for the disease model. In solving the given problem, piecewise Newton polynomial approximation is the used. In the following, piecewise fractional hybrid operators are shown to be essential for the efficient numerical solution of the syphilis infection model due to their closer ability to mimic the change in systems dynamics during transition from one phase to another. Data aided control design at various fractional order values is made possible by the use of Newton polynomial interpolation method hence allowing the systems behavior to be approximated and solved in a flexible and efficient manner. It makes it possible for researchers to accurately record these phases involving circulation, transmission and distribution of syphilis thereby improving on the kind of interventions that policy makers can take in phases of transmission. Several simulations have been presented here on the basis of suggested model for different fractional orders which is supported by the fact that the performance of the piecewise operators are optimum if compared separately with the fractional and classical operators. It allows giving thorough consideration to the stability, perturbation and the critical point showing useful qualitative insights into the management of instabilities resulting from continual shocks in system dynamics and a solid groundwork for constructing the bette strategies for stabilizing syphilis transmission dynamics and furthering the public health objectives. This also suggests that a time interval can be partitioned into two or more intervals to settle instability in a number of phenomena in the global context in which sudden alterations towards the dynamics of different densities exist. When fractional orders are smaller, the control approach works better, as the use of smaller fractional orders enhances the efficiency of control strategies by refining predictive capabilities, thereby guiding the development of more targeted and effective interventions. Thus, the suggested approach is crucial for studying epidemiological models. Even so, some disease model dynamics exhibit stochastic behavior. The developed epidemic model using piecewise fractional order derivatives reveals that syphilis transmission dynamics and treatment effects vary significantly across different time phases and between sexes, with early detection and gender-specific interventions being crucial for effective management. The model emphasizes the rapid initial spread of syphilis, underscores the significance of addressing undetected latent infections, and highlights the crucial role of timely and compliant treatment in reducing transmission and preventing antibiotic resistance.

Accurately estimating model parameters like transmission rates, recovery rates, and fractional orders from real-world data is challenging. Incorrect parameter estimation can lead to inaccurate model predictions, compromising the model’s reliability and its effectiveness in guiding public health interventions. Develop robust techniques for parameter estimation that can effectively manage the uncertainties present in real-world data. By capturing the complex, non-linear dynamics of the disease, the approach provides a detailed understanding that informs more effective and targeted public health strategies.
